# *In vivo* partial reprogramming by bacteria promotes adult liver organ growth without fibrosis and tumorigenesis

**DOI:** 10.1016/j.xcrm.2022.100820

**Published:** 2022-11-15

**Authors:** Samuel Hess, Timothy J. Kendall, Maria Pena, Keitaro Yamane, Daniel Soong, Linda Adams, Richard Truman, Anura Rambukkana

**Affiliations:** 1 Institute for Regeneration and Repair, The University of Edinburgh, Edinburgh, UK; 2 Centre for Regenerative Medicine, The University of Edinburgh, Edinburgh, UK; 3 Centre for Inflammation Research, The University of Edinburgh, Edinburgh, UK; 4 Edinburgh Pathology, The University of Edinburgh, Edinburgh, UK; 5 US Department of Health and Human Services, Health Resources and Services Administration, Healthcare Systems Bureau, National Hansen’s Disease Program, Baton Rouge, LA, USA; 6 Medical Research Council Centre for Reproductive Health, The University of Edinburgh, Edinburgh, UK; 7 Department of Pathobiological Sciences, School of Veterinary Medicine, Louisiana State University, Baton Rouge, LA, USA; 8 Edinburgh Infectious Diseases, The University of Edinburgh, Edinburgh, UK; 9 Centre for Discovery Brain Sciences, The University of Edinburgh, Edinburgh, UK

**Keywords:** organ growth, liver regeneration, partial reprogramming, rejuvenation, aging, machine learning, stem cells, regenerative medicine, *Mycobacterium leprae*, nine-banded armadillo

## Abstract

Ideal therapies for regenerative medicine or healthy aging require healthy organ growth and rejuvenation, but no organ-level approach is currently available. Using *Mycobacterium leprae* (ML) with natural partial cellular reprogramming capacity and its animal host nine-banded armadillos, we present an evolutionarily refined model of adult liver growth and regeneration. In infected armadillos, ML reprogram the entire liver and significantly increase total liver/body weight ratio by increasing healthy liver lobules, including hepatocyte proliferation and proportionate expansion of vasculature, and biliary systems. ML-infected livers are microarchitecturally and functionally normal without damage, fibrosis, or tumorigenesis. Bacteria-induced reprogramming reactivates liver progenitor/developmental/fetal genes and upregulates growth-, metabolism-, and anti-aging-associated markers with minimal change in senescence and tumorigenic genes, suggesting bacterial hijacking of homeostatic, regeneration pathways to promote *de novo* organogenesis. This may facilitate the unraveling of endogenous pathways that effectively and safely re-engage liver organ growth, with broad therapeutic implications including organ regeneration and rejuvenation.

## Highlights

• ML infection of adult nine-banded armadillos promotes *in vivo* liver growth
• Enlarged infected livers are functionally and architecturally normal without tumors
• ML-induced partial reprogramming to progenitor/regeneration state drives liver growth
• ML bypassing of normal liver growth restriction offers safer repair interventions

## Graphical abstract

**Figure:**
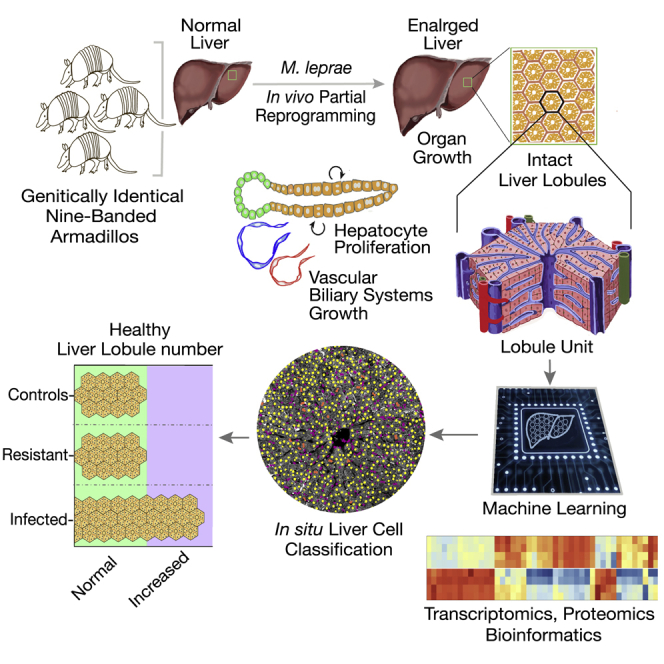

## Teaser

Organ-level tissue regenerative and rejuvenation approaches are currently not available. Using an evolutionarily refined infection model of nine-banded armadillos and *Mycobacterium leprae*, Hess et al. show that bacteria-induced *in vivo* partial reprogramming of liver cells into a regenerative state drives functionally and architecturally normal adult liver organ growth without fibrosis or tumorigenesis.

## Introduction

Adult organ growth promotion and rejuvenation are idealized strategies for treating dysfunction in disease, injury, or aging.^1^^,^^2^^,^^3^ Such strategies must engage highly coordinated multilineage functions *in vivo*. Although *in vitro* models, organoids, and mini-organs have potential for drug discovery, disease modeling, and regenerative medicine,^4^^,^^5^ they fail to model required organ-level complexity. Consequently, despite advances in such approaches,^2^^,^^5^ no current strategy achieves effective regrowth or rejuvenation of adult organs in chronic or aging-associated human diseases.

Liver is the exemplar organ for studying growth and regeneration.^6^^,^^7^ Unlike other solid organs, adult liver has the capacity to regain the prior mass after tissue loss, restoring homeostasis.^7^ In human chronic liver disease, repeated inflammatory injury and parenchymal cell death stimulate regenerative restitution of the liver cell mass in parallel with a wound-healing response.^8^ Some regenerative capacity remains in cirrhosis, although complete recovery is impossible and transplantation remains the only treatment. Chronic injuries are associated with increased risk for malignancy, which is highest in chronic viral infections.^9^^,^^10^ Endogenous pathways regenerating damaged liver remain poorly characterized, and failures of understanding contribute to a lack of pro-regenerative clinical strategies. As the health and economic burden of liver diseases rapidly increase,^11^^,^^12^ the absence of such repair strategies is critical. Moreover, the aging liver is more prone to progressive diseases as physiological functions decline.^13^^,^^14^^,^^15^ Maintenance of healthy liver for healthy aging is critical because it directly or indirectly influences other organ function, but there are no rejuvenating strategies slowing or reversing declining liver function during aging.

Current study of liver regeneration uses short-lived rodent models that require hepatocyte loss to stimulate regeneration^8^^,^^16^^,^^17^ that ceases when the original liver size is reached. Mechanisms stopping the response once the prior organ size is reached are unknown.^17^ The ability to bypass such upper-limit restriction would allow regeneration to be studied without prior liver injury. Understanding how regenerative machinery can be engaged *de novo* will provide paradigm-shifting adult organ regrowth and rejuvenation clinical strategies that could reduce or replace transplantation, but no such *in vivo* model is currently available.

Recent studies using the overexpression of the OSKM factors (Oct4, Sox2, Klf4, c-Myc) that originally generated induced pluripotent stem cells (iPSCs) from somatic cells^18^^,^^19^^,^^20^^,^^21^^,^^22^^,^^23^ showed a proof-of-principle that resetting committed cells to a progenitor stage of the same lineage permits tissue regeneration and rejuvenation. Therefore, alternative approaches that potentially increase adult tissue plasticity, proliferation, and de-differentiation should also be explored as strategies for tissue rejuvenation and regeneration.

Our studies on the biology of *Mycobacterium leprae* (ML)-host interaction^24^^,^^25^^,^^26^^,^^27^ led to the identification of ML’s natural ability to hijack the plasticity and regenerative properties of adult Schwann cells, partially reprogramming them into a progenitor cell/stem cell state beneficial to the bacteria.^28^ At the host level, ML-induced reprogramming promotes growth of infected tissues permitting bacterial propagation.^24^^,^^25^^,^^28^^,^^29^ These host-dependent features of ML without cytopathic or adverse effects during the establishment phase of infection permit use of ML as an evolutionarily adapted bacterial model for dissecting undefined host endogenous pathways.^29^^,^^30^^,^^31^^,^^32^^,^^33^

Nine-banded armadillos (*Dasypus novemcinctus*) are New World placental mammals, the only mammal to produce four genetically identical/clonal litters and a natural host of ML.^33^^,^^34^^,^^35^ Experimental inoculation with viable ML produces disseminated infection,^33^^,^^34^^,^^35^^,^^36^^,^^37^ and their lifespan (12–13 years in the wild, up to 20 years in captivity) and core body temperature (32°C–35°C) are optimal for *in vivo* ML replication.^33^^,^^34^^,^^35^ Since their discovery as a natural host of ML,^35^ armadillos have been used for *in vivo* propagation of ML in the liver for harvesting bacteria for research.^36^ We explored whether this co-evolved bacterial pathogen in the liver of susceptible hosts exploited the same reprogramming strategies to expand host cells *in vivo* during natural infection as those observed *in vitro* in adult Schwann cells.^28^

We report a natural *in vivo* model of ML-infected nine-banded armadillos for mammalian adult liver growth at organ level without prior injury. We showed that bacteria-induced *in vivo* partial reprogramming significantly increased liver size with sustained function and architecture but without damage, fibrosis, or tumorigenesis during the establishment phase of infection. We define which cell types promote this organ growth and show that healthy liver lobule number, not size, with a proportionate expansion of the hepatocyte mass and vascular and bile ductal systems, is responsible. We delineate the molecular details to show evidence that ML have adapted dynamic partial reprogramming, regenerative, and developmental/fetal mechanisms to promote *de novo* liver organogenesis while maintaining tissue-protective and tumor-preventive strategies.

## Results

### *In vivo* ML infection of nine-banded armadillos promotes organ growth

**Figure 1:**
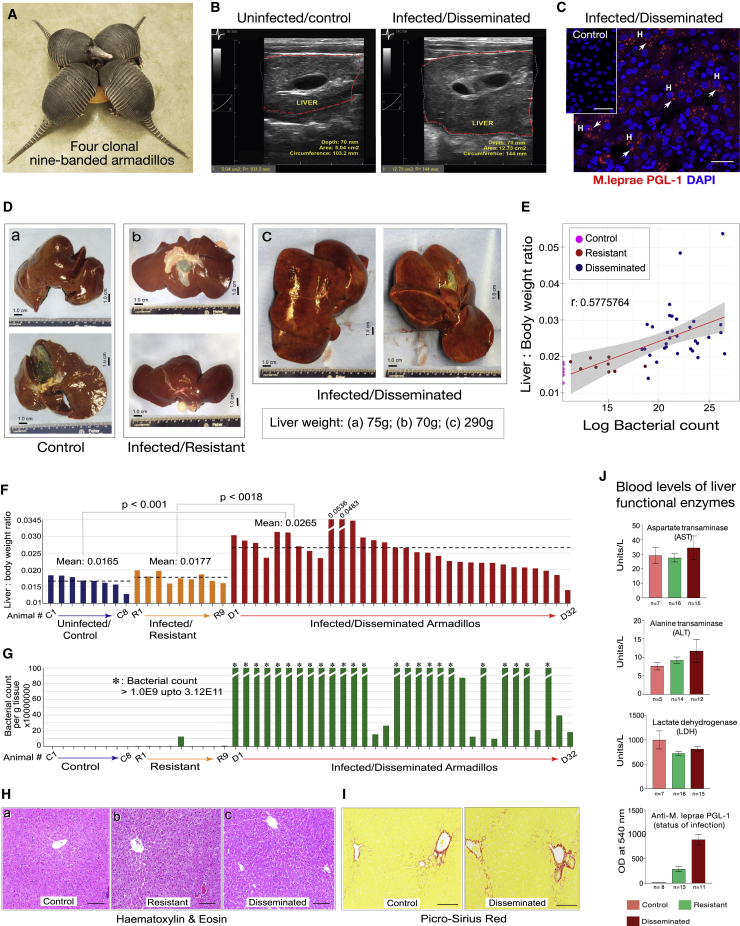
*Mycobacterium leprae* (ML) promote *in vivo* organ growth of normal liver in adult nine-banded armadillos without hepatic damage (A) Nine-banded armadillos produce litters of four genetically identical offspring. (B) The parenchyma of all animals has identical echotexture, but livers of infected animals are enlarged (right) compared with uninfected animals (left). (C) Confocal images show ML-laden hepatocytes (*H*) in infected liver detected by ML-specific PGL-1 antibody (red, arrowheads; blue, DAPI). Inset: control liver (scale bars, 40 μm; also see Figure S1). (D) Eviscerated livers of (a) control, (b) resistant, and (c) infected (19-month example) animals show identical lobation with smooth, uniform outer capsules; organ weight is indicated. Scale in field. (E) Scatterplot of bacterial counts versus liver/body weight ratios for armadillos presented in (F) and (G), with co-efficient of correlation (r), best-fit linear line (red) with confidence interval (gray) (rs = 0.5775764, p = 0.00007687). (F) Liver/body weight ratios of uninfected (blue), resistant (yellow), and infected animals (red). (G) Bacterial counts (per gram of tissue) in livers from the same control, resistant, and infected animals presented in (F). (H) The parenchyma of infected livers (c) is identical to that of uninfected (a) and resistant (b) animals without damage (H&E-stained sections; scale bars, 200 μm). (I) There is no scarring in infected (right), uninfected (left), or resistant animals (picrosirius red [PSR]-stained sections; scale bars, 200 μm; also see Figure S2). (J) Serum levels of liver functional enzymes AST, ALT, and LDH, as well as serum ML-PGL-1 antibody levels, mean + SD from indicated number of infected armadillos during the establishment phase of infection compared with controls; anti-PGL level, p < 0.0001 (n = number of animals in each group with available test result).

Adult nine-banded armadillo (>1.5–2 years old) livers with disseminated infection (“infected”) after injection of viable ML were compared with those from animals resistant to infection (“resistant”) and uninfected animals (Figures 1 and S1; Table S1; STAR Methods). During natural infection, ∼95% of humans and 20% of armadillos clear ML immediately, whereas infection progresses in the remainder. Clonal armadillo siblings (Figure 1A) were either fully resistant to infection or showed disseminated infection, indicating a strong heritable component for susceptibility and clearance. Resistant animals showed initial responses to infection, determined by serum ML-specific phenolic glycolipid-1 (PGL-1) antibody levels (Figure S1C; STAR Methods), but bacteria failed to propagate in the liver and only a few, presumably non-viable or dead, bacilli remained (Figure 1G; Table S1). Total liver/body weight ratio was significantly increased in armadillos infected for a period of 10–30 months compared with resistant (p < 0.0018) or uninfected animals (p < 0.001) (Figures 1B, 1D, and 1F). Livers from most infected animals showed a high bacterial count (up to 3.0E11 bacilli/g) (Figures 1C, 1F, 1G, and S1; Table S1). Liver/body weight ratio correlated with hepatic bacterial load in infected animals (Spearman's rho [rs] = 0.5775764, p = 0.00007687; Figure 1E). Immunolabeling with an ML-specific anti-PGL-1 antibody and Wade-Fite acid-fast mycobacterial staining^28^^,^^38^ revealed ML in most hepatocytes and macrophages in small granulomas in infected animals (Figures 1C and S1D).

Armadillos comprised clonal animals and wild-born or captive-born animals (Figure 1A; Table S1). Clonal infected animals showed almost identical liver mass increases and bacterial counts (Figure S1), but there was no difference in these metrics between wild- or captive-born animals or between males and females (Table S1). The influence of non-ML infection was excluded by clearance of prior infection with antibiotic, antifungal, and antiparasitic drug treatment for >1 year in captivity prior to inoculation. Identical treatment of resistant and uninfected animals did not induce liver growth. Therefore, we concluded that ML-induced liver growth is unique and specific.

### Enlarged infected livers have intact architecture and vascular organization without damage, fibrosis, steatosis, or tumor formation

*In vivo* ultrasonographic assessment demonstrated identical liver echotexture and normal lobation in all groups; livers of infected animals were enlarged (Figure 1B). Macroscopic examination confirmed identical lobation in all groups, with smooth, uniform capsules (Figures 1D, S1A, and S1B). Livers from infected animals were larger, but all lobes were enlarged similarly, and their relative proportions were normal (Figures 1D, S1A, and S1B). On sectioning, the parenchyma of livers from each group was identical. No masses were present in livers of any animal (Figures 1D, S1A, and S1B).

**Figure 2:**
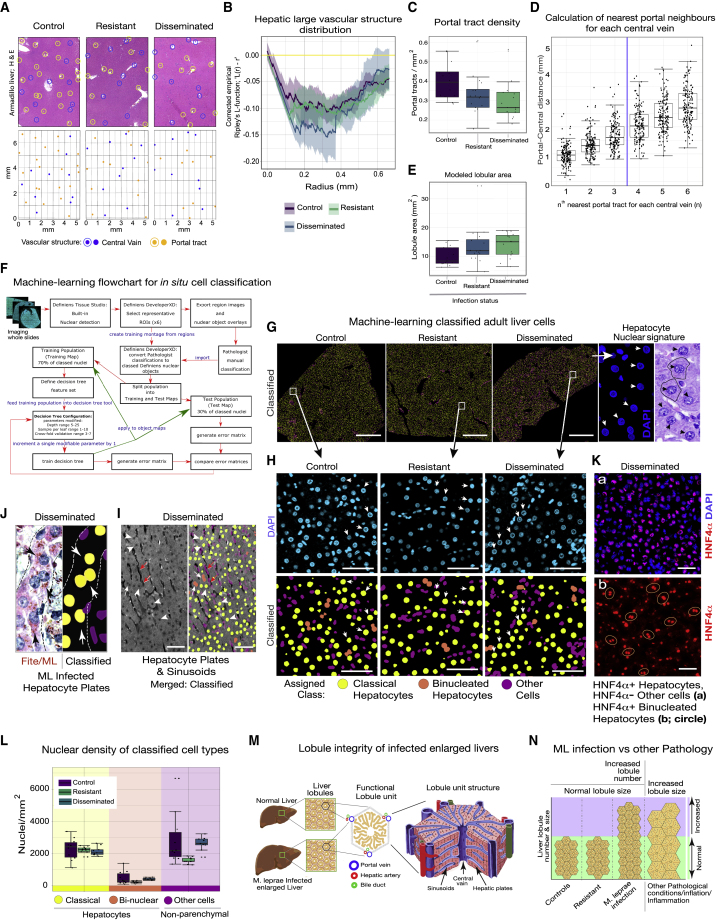
Bacteria-induced *in vivo* liver organ growth maintains normal hepatic lobular and vascular microarchitecture (A) Portal tracts and central veins were annotated on digital images of H&E-stained sections (upper) and spatial point patterns (lower) created (representative images, scale shown in lower panels; also see Figure S2). (B) Vascular structures in all groups (n = 13 control, 16 resistant, and 15 disseminated) were similarly dispersed without loss of regular arrangement in infected animals (plots of the empirical corrected Ripley’s L-function for each group fall below the yellow line representing complete spatial randomness, indicating consistent regularity of vascular structures in all groups). (C) Portal tract density in 2D images was significantly reduced in infected animals compared with control animals, suggesting branch elongation of the biliary fractal geometry (group sizes as in B). Individual points and median (center line), first and third quartiles (lower and upper box limits), 1.5 × interquartile range (whiskers); same error bars are applied to (D), (E), and (L) below. (D) The largest “step” in mean distance in the 6 nearest portal neighbors for every central vein in 13 control animals was between the 3rd and 4th nearest neighbor. (E) Mean modeled lobule size applying the lobule-as-hexagon paradigm, with the radius derived from the mean of the nearest three portal tracts for each central vein, showed individual lobular size was not increased in the larger livers of infected animals (group sizes as in B). (F) Machine-learning workflow for cell classification. (G) Representative examples of whole sections visible using autofluorescence for cell-type classification (scale bars, 1 mm). Nuclear signature of hepatocytes (DAPI) and histology of liver sections for cell classification are shown in the far right panels (arrowheads). (H) DAPI-stained nuclei (top) with matched class (bottom) showing classification accuracy of hepatocytes and non-parenchymal cells (scale bars, 50 μm). (I) Representative field from an infected animal showing intact hepatic plates and sinusoidal architecture using autofluorescence (left panel) overlaid with cell-type classification (right panel). Spatial distribution of classified classical hepatocytes (yellow), binucleated hepatocytes (orange), and other (non-parenchymal) cells (purple). Scale bars, 50 μm. (J) Superimposed Fite bacteria-stained image of infected liver (left) with classified classical hepatocytes (right) in hepatocyte plates (dotted lines) harboring ML (arrows). (K) Infected liver labeled with hepatocyte nuclear factor 4 alpha (HNF4α) antibody showing classified classical hepatocytes (a) and binucleate hepatocytes (b; circled) corresponding to HNF4α^+^ hepatocytes (scale bars, 50 μm). (L) Density of classical hepatocytes in enlarged livers of infected animals was the same as in uninfected and resistant livers (n = 10 control, 10 resistant, and 9 disseminated). (M) Schematic showing significantly enlarged livers that have lobules of normal size and normal classical hepatocyte densities, reflecting “normal” organ growth maintaining normal liver microarchitectural and lobule organization by including matched generative activity from all native cell types and structures. (N) Liver lobules are increased in number, but not in size, in infected armadillo livers contributing to organ growth, in contrast with pathologically inflated livers with increased liver lobule size.

**Figure 3:**
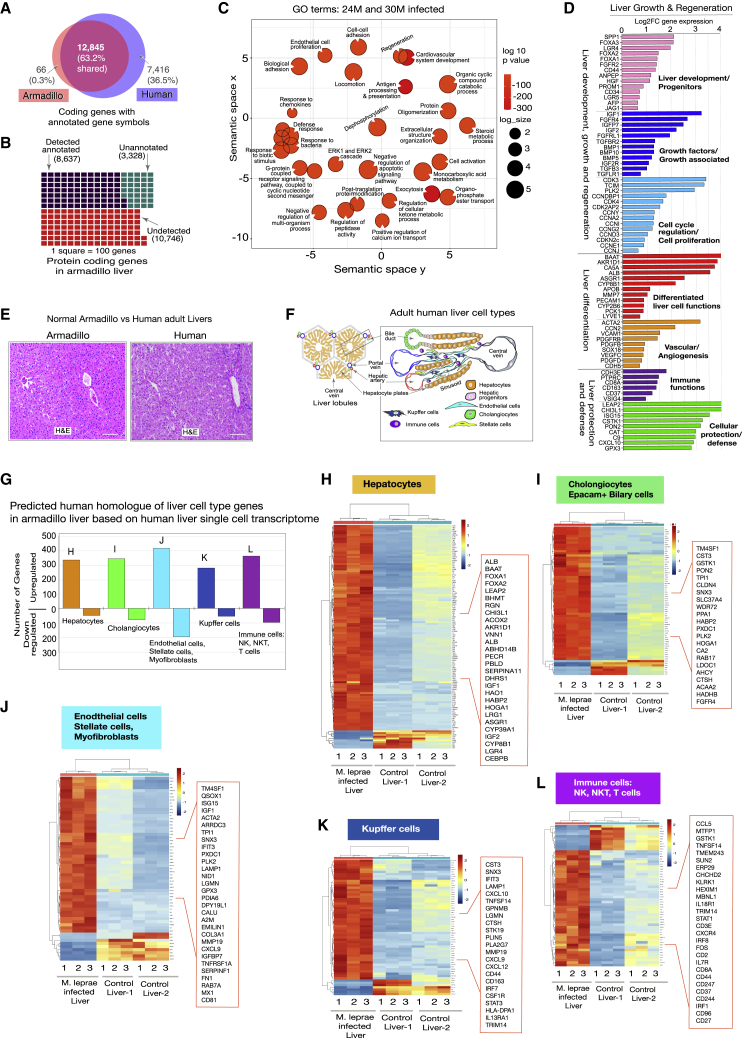
The transcriptome of infected nine-banded armadillo livers reflects organ regeneration and reactivation of liver developmental progenitors, growth, and differentiation (A) Shared unique annotations for nine-banded armadillo and human protein-coding genes with designated gene symbols, showing significant overlap between nine-banded armadillo and human. (B) Waffle diagram of RNA-seq-detected annotated (purple), RNA-seq-detected unannotated (green), and undetected (red) genes out of the total known armadillo protein-coding genes. (C) GO terms of functional categories of significantly upregulated genes in infected armadillo liver identified by gene set enrichment analysis (GSEA) in 2D semantic space, with related terms positioned closer together. (D) Selected upregulated common genes in infected livers are grouped into indicated functional categories related to liver growth and regeneration. (E) Representative H&E staining showing similarities of armadillo (left) and human (right) liver (scale bars, 200 μm). (F) Schematic of liver lobular structure and major cell types in adult human liver, resembling armadillo liver cell types indicated in (E). (G) Cross-referencing of differentially expressed genes from infected armadillo livers with annotated liver cell types from published scRNA-seq from normal adult human liver^39^ showing total gene number for each cell type. (H–L) Selection of differentially expressed genes (p < 0.05, fold change > 1.5) in heatmaps for indicated individual liver cell types in infected armadillo livers based on human gene homologs in corresponding human liver cell types identified by scRNA-seq; highly upregulated and functionally relevant selected genes in each cell type are shown in boxed areas. Expression is shown as log fragments per kilobase of exon per million mapped fragments (FPKM); genes are hierarchically clustered according to expression values.

**Figure 4:**
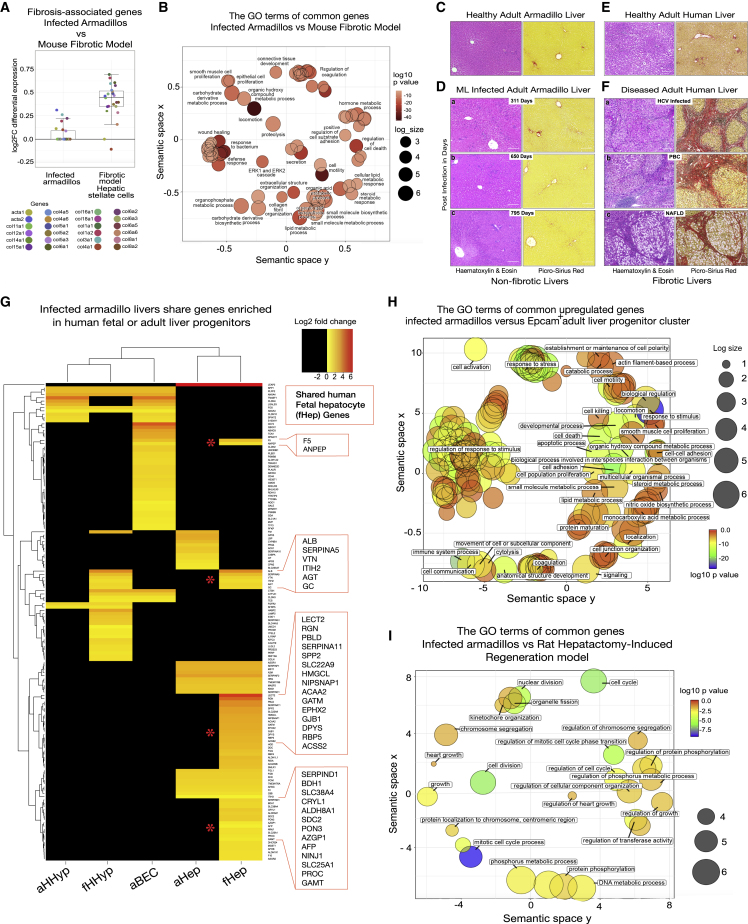
ML-infected adult armadillo livers are non-fibrotic but pro-regenerative: comparison with human fibrotic liver diseases, human fetal livers, and rodent fibrotic and hepatectomy-induced regeneration models (A) Upregulation of collagens and smooth muscle actin is restricted in non-fibrotic livers of infected armadillo liver compared with the common suite of collagens produced in pro-fibrotic activation of scar-orchestrating cells in mouse fibrotic model. Individual points and median (center line), first and third quartiles (lower and upper box limits), 1.5 × interquartile range (whiskers). (B) GO terms of common genes upregulated in both the unscarred infected livers and a discrete lineage of scar-orchestrating hepatic stellate cells in a murine model of fibrogenesis induced by chronic injury with carbon tetrachloride includes regenerative, homeostatic, and metabolic processes in addition to wounding responses and extracellular matrix production. (C and D) Uninfected armadillo liver (C) has the same sinusoidal organization as the liver of animals infected with ML (D), and increasing duration of infection (up to 795 days) does not induce fibrosis (Da–c, PSR). Scale bars, 200 μm. (E and F) Organization of normal adult human liver (E) shows no difference from normal adult nine-banded armadillo liver (C) (H&E and PSR). However, normal architecture is lost in human chronic liver diseases (F): (Fa) chronic infection with hepatitis C virus (HCV); (Fb) granulomatous injury (primary biliary cholangitis [PBC]); and (Fc) metabolic injury with lipid accumulation, non-alcoholic fatty liver disease (NAFLD), all leading to extensive scarring and loss of normal vascular relationships representing cirrhosis (scale bars, 200 μm). (G) Infected armadillos show upregulation of genes included in sets of hepatic and biliary genes that define populations of fetal and adult hepatocytes (fHep and aHep), fetal and adult hepatobiliary hybrid progenitors (fHHyP and aHHyP), and adult biliary epithelial cells (aBECs) in human liver.^40^ Heatmap shows log2 fold change upregulation in infected livers for genes appears in one or more gene sets used to define epithelial or progenitor cell types. Some genes appear on more than one cell-type-defining list; black entries for a stated cell type indicate where the gene does not appear on that cell-type-specific list. Selected genes from clusters specifically shared with human fetal liver (indicated by asterisks) are shown (inset) (see Data S2). (H) GO terms of common genes upregulated in both ML-infected livers and defining the EPCAM1^+^ progenitor cluster in adult human liver.^39^ (I) GO terms of common genes upregulated in both ML-infected livers and the livers of rats 24 h after partial hepatectomy^41^ include those relating to DNA synthesis, subcellular organization, and other proliferation-related terms.

ML were demonstrable in the cytoplasm of cells with characteristic hepatocellular nuclear features and distribution (Figures 1C and S1D). Blinded histological examination of random parenchymal blocks revealed that the portal-central vascular relationships of normal mammalian liver were present in the livers of infected animals (Figures 2A, 3E, 4C, 4E, and S2). Hepatocytes were present in single-cell plates, separated by sinusoidal vascular channels (Figures 1H, 1I, 2A–2G, and S4). Infected livers showed variable inflammation and irregularly distributed small non-necrotizing granulomas consisting mostly of bacteria-laden macrophages (Figures S1A–S1C and S10). Focal, pericentral cell-plate twinning was present in a single infected animal, suggesting regenerative activity (Figure S5).

There was no other abnormality in infected livers (Figures 1H, 4C, 4E, S1, S2, and S5). Specifically, there was no hepatocellular ballooning, steatosis, or cell death (apoptotic acidophil bodies, necroinflammatory foci, or TUNEL-positive cells), or evidence of prior cell death (“ceroid-laden” macrophages). There was no portal or parenchymal edema, no sinusoidal dilation, and no nodularity. There was no dysplasia in the livers of any animal (Figures 1H, 4B, 4C, and 4E). There was no scarring demonstrable on sensitive extracellular matrix (ECM) stains (Figures 1I and 4B). The inflammatory injury, steatosis, and scarring seen in chronic human inflammatory diseases as a result of chronic viral infection, autoimmune injury, or metabolic injury (Figure 4F) were not present in infected armadillo livers. Serum levels of the liver functional enzymes aspartate transaminase (AST), alanine transaminase (ALT), and lactate dehydrogenase (LDH) were not deranged in infected animals during the early stage of bacterial propagation, while serum antibody responses to ML remained high, indicating the absence of hepatocellular injury during infection-induced liver growth (Figure 1J).

### Architectural integrity of infected enlarged livers

To quantitatively evaluate the architectural integrity of infected liver, we annotated positions of portal tracts and central veins on images of H&E-stained sections (examples from 10 animals in each group are shown in Figure S2) and created spatial point patterns (Figure 2A). The distribution of all annotated vascular structures was quantified, and groupwise analysis demonstrated no differences (studentized permutation test for grouped point patterns, studentized permutation test of Hahn statistic [999 random permutations] = 1.6353, p = 0.549); in all groups, large vascular structures were evenly dispersed (empirical function plots below the yellow line that represents complete spatial randomness), and there was no loss of the regular arrangement in the disseminated animals (Figure 2B). To understand the fractal geometry of the biliary tree in the enlarged livers, we calculated the intensity of portal tracts in the 2D sections. The portal density in the livers of animals with disseminated infection was significantly different by one-way ANOVA (F(2,41) = 4.115, p = 0.0235); *post hoc* comparison using Tukey’s HSD (honestly significant difference) test indicated that the mean density in infected animals was less than that of the control animals (p = 0.01999), suggesting elongation of the individual branches in addition to any branching occurring during liver growth (Figure 2D).

### Proportionate lobular expansion in infected enlarged livers

To model the cross-sectional representation of functional lobular units of the liver, we calculated nearest-neighbor distances between portal tracts and central veins to allow lobule area to be determined, based on the hexagonal paradigm often used to represent the polygonal lobules with between three and seven faces in mammals.^42^ Polygonal liver lobules most commonly have portal tracts at three apices, although this, too, is variable. To determine whether this assumption could be used to calculate a mean value for the radius of each lobule, we calculated the nearest six portal tract neighbors to each separate central vein profile in control animals (Figure 2C). The greatest difference between the mean of the k and k+1 neighbors was at the k = 3:4 boundary, indicating that the nearest three portal tracts were segregated from the next nearest portal tracts in armadillo liver, and the mean of these values for each central vein was used to calculate modeled lobule size. The size of individual lobules was not significantly different between groups by one-way ANOVA (F(2,41) = 2.702, p = 0.079; Figure 2E). These findings collectively indicate no microscopic abnormality in infected livers despite long-term ML infection and organ enlargement, suggesting ML-induced liver organ growth involves proportionate vascular, biliary, and lobular growth.

### Increased hepatocyte mass in ML-induced liver growth

vTo assess which liver cell type(s) contributes to ML-induced liver growth, we quantified the cellular composition of liver sections using machine-learning cell classification (Figure 2F; STAR Methods; Table S2). All cells in DAPI-stained liver sections were classified based on nuclear morphology as “classical hepatocytes” with large spherical nuclei (Figures 2G and 2H), “binucleated hepatocytes” (Figures 2H and 2I), or non-hepatocytes (“other”) using a manually trained machine-learning classifier (Figures 2H–2J and S2–S4). Although some errors are apparent in these representative regions, they are in the expected range (Table S2) considered valid for analysis across whole sections. Accuracy of classification is supported by the localization of hepatocytes (in yellow) within hepatocyte plates (Figures 2H, S3, S4, and S8E) and the localization of purple-labeled “non-hepatocytes/other cells” within vessel walls, granulomas, and sinusoids (Figures S3, S4, and S8E).

Confirmation was provided by labeling with a cross-species (mouse/human) antibody for the hepatocyte-specific transcription factor (TF) HNF4α (Figures 2K and S8E). This demonstrated that HNF4α immunopositivity correlated with classified single or binucleated hepatocytes; there was no HNF4α immunopositivity in nuclei of cells classified as ‘‘non-hepatocytes/other.’’ The normal sinusoidal architecture in livers of animals with disseminated infection was visible from the normal hepatic autofluorescence (Figures 2I and S4).

Using this classification, we calculated the density of nuclei of each cell class. The mean density of classical hepatocytes in each group was not significantly different by one-way ANOVA (F(2,26) = 0.185, p = 0.832; Figure 2K). There were more binucleate hepatocytes in infected livers compared with controls, although the mean number for each group did not differ significantly by one-way ANOVA (F(2,26) = 3.112, p = 0.0614). There were also more “other” cells in infected livers of infected animals, reflecting variable inflammation and focal small granulomas; although the mean number was significantly different by one-way ANOVA (F(2,26) = 4.653, p = 0.0187), there was no significant difference between the mean number of “other” cells in control and infected animals by *post hoc* Tukey’s HSD testing (p = 0.1349992) (Figure 2K), suggesting that “other” cells, which mostly comprise immune and endothelial cells, are not responsible for the increased parenchymal mass in infected livers. These combined analyses show that the microarchitectural lobular organization and hepatocellular composition of infected enlarged livers were normal, in keeping with the macroscopic and histopathological normality (Figures 2 and S4). The enlarged liver in disseminated ML infection is not a consequence of pathological “inflation” of lobules, which would be associated with a decrease in hepatocellular density but shows lobules of normal size and with normal hepatocyte density, indicating “normal” organ growth and proportionate expansion of vascular and ductal systems promoted by ML (Figures 2L–2N).

### RNA sequencing of ML-infected livers with human gene annotation reveals large transcriptional changes reflecting partial hepatocellular reprogramming

RNA sequencing (RNA-seq) was undertaken to define the molecular signature underlying ML-induced liver growth. Among protein-coding genes, 99.5% of those with gene symbols are shared with annotated human genes, so known mammalian gene functions were used to interpret armadillo RNA-seq data (Figures 3A–3C; STAR Methods). Gene Ontology (GO) analysis of differentially upregulated genes in infected livers demonstrated upregulation of cellular activation, progenitor markers, and metabolic processes that conceptually connect to increased liver cell mass (Figures 3 and 4). Enrichment of “Regeneration,” “Homeostatic process,” and “Wound healing” further suggests that the liver growth could involve endogenous regeneration, homeostasis, and repair pathways (Figures 3C, 3D, 6A, and 6B). Enrichment of genes associated with GO terms for vascular development, angiogenesis, and bile duct formation-related processes and upregulation of numerous ECM and collagen genes are in keeping with expansion of vascular and biliary systems and the generation of supportive matrix for these newly formed vascular and ductal structures (Figures 3C, 3D, and S4). Selected genes related to liver development, growth, cell-cycle progression, and regeneration (based on mouse and human studies) are shown in Figure 3D.

### All normal liver cell types contribute to the growth of infected armadillo livers based on predicted similarities to human liver single-cell transcriptomes

To investigate the contribution of different cell types, based on their gene expression, to ML-induced liver growth, we cross-referenced differentially expressed armadillo genes with human liver single-cell RNA-seq (scRNA-seq) data.^39^ ML-induced genes are associated with all key liver cell types in armadillos (Figures 3H–3L; Data S1), in keeping with proportional functional contribution to liver growth at the organ level.

### ML-infected enlarged livers are pro-regenerative but non-fibrogenic

Next, we compared transcriptional features of ML-infected armadillo liver with rodent fibrotic and regenerative models, human liver diseases, and human fetal liver. In adult livers, upregulation of ECM markers is associated with fibrosis.^8^^,^^43^ When compared with our previously described mouse model of fibrosis,^43^ we found that the transcriptional response in infected liver overlaps with that seen after activation of quiescent hepatic stellate cells into myofibroblasts responsible for ECM production and organization, a shared effector event in fibrotic liver disease^43^ (Figures S7B and S7C). However, the number of genes coding for different collagen species upregulated, and the extent of upregulation, is muted in the unscarred livers of ML-infected animals compared with the profibrotic response to injury in the mouse fibrosis model (Figures 4A and 4B), in keeping with the absence of histological fibrosis (Figures 1H, 1I, and 4C).

To further support the hypothesis that ML infection induces a liver progenitor-like state *in vivo* and demonstrate potential relevance of our findings to human liver regeneration, we compared the transcriptome of infected armadillo livers with adult and fetal hepatic progenitors or related populations defined from scRNA-seq analysis of human liver.^39^^,^^44^^,^^40^ Despite the difference in species, technology, and biological context, a signal attributable to hepatocyte progenitors was clear. A range of fetal and adult liver progenitor-like markers is upregulated in the livers of infected armadillo livers, suggesting *in vivo* generation of progenitor populations. Livers of infected armadillos show upregulation of genes whose expression defines populations of fetal (fHep) and adult hepatocytes, fetal (fHHyP) and adult hepatobiliary hybrid progenitors, and adult biliary epithelial cells in human liver (Figure 4G),^40^ including the fHep marker AFP and the progenitor markers PROM1 and FGFR2. The shared genes upregulated in infected armadillo livers and those defining fHHyP and fHep map to GO terms such as wound-healing responses and metabolic processes.

Importantly, there is also upregulation in infected armadillo livers of 477 markers of epithelial cell adhesion molecule (EPCAM1)^+^ progenitor populations in adult human liver defined by Aizarani et al.^39^ (including PROM1, SFRP5, CLDN3, CLDN4, CLDN10, and ANXA4), markers of a central population of uncommitted bipotential epithelial progenitor cells (SFRP5 and FGFR2), and markers defining both hepatocyte-biased (ALB, SERPINF1, and FGB) and cholangiocyte-biased progenitors (ANXA2, BIRC3, and TM4SF1). The GO terms significantly mapped to the shared genes upregulated in ML-infected armadillo liver and defining the EPCAM^+^ progenitor cluster from normal adult human liver (cluster 4) are shown in Figure 4H, and the full g:Profiler report is provided in Data S2.

Furthermore, MacParland et al.^44^ determined differential gene expression in AFP-positive versus AFP-negative cells, proposing that AFP-positive cells throughout lobules in adult liver represent a heterogenous population of hepatic progenitor cells; there were 233 shared genes upregulated in AFP-positive versus AFP-negative cells and in infected armadillo livers, and the GO term mapping of these included microRNA (miRNA) and organonitrogen metabolic processes. A total of 144 shared genes were downregulated in the same comparisons, mapping to RNA biosynthetic and metabolic processes and chromatin organization. The complete g:Profiler reports of GO term mappings using the sets of shared upregulated or downregulated genes are shown in Data S2.

### ML reactivates liver regeneration-associated genes similar to the rat hepatectomy model

In a rat partial hepatectomy model, a model of rapid liver regeneration,^41^ there were 60 upregulated genes also upregulated in ML-infected armadillo livers. These shared upregulated genes map to GO terms related to cell division and subcellular organization (Figure 4H). Only 17 genes were equivalently downregulated in both model systems.

### ML infection selectively induces trophic factors/pathways while preserving liver functional markers

**Figure 5:**
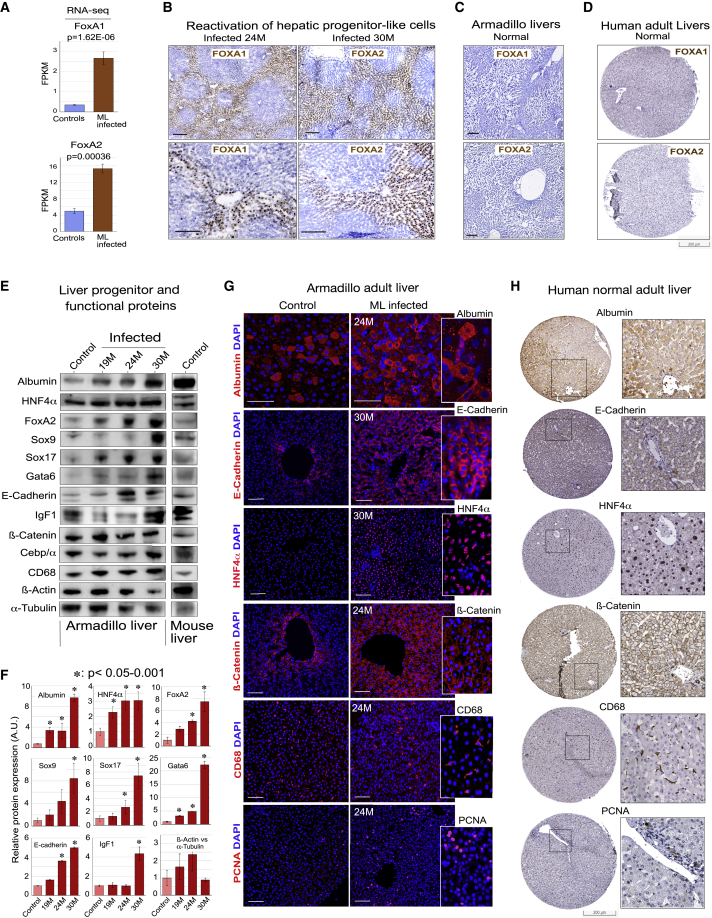
Reactivation of liver progenitor/development marker proteins with sustained liver functions in infected livers (A) FOXA1 and FOXA2 genes are significantly upregulated in infected livers. Mean ± SD from triplicates from two infected and two control armadillos. (B–D) FOXA1 and FOXA2 proteins are reactivated specifically in selected pericentral/midzonal hepatocytes in infected livers *in situ* (B) but are absent in uninfected adult armadillo (C) and normal human liver (D) (human liver images from Human Protein Atlas). (E) Reactivity of cross-species (mouse/human) antibodies to liver progenitor and other functional markers with total armadillo liver extracts by western blotting shows upregulation in infected liver. Mouse adult liver (right panel) as positive control. (F) Quantification of protein expression (normalized with beta-actin and alpha-tubulin) for proteins (E). Mean ± SD from triplicates from three infected and two control armadillos. (G) Confocal immunofluorescent imaging of selected upregulated proteins indicative of functional liver (albumin) and developmental or homeostatic mature hepatocyte activity (E-cadherin, ß-catenin, HNF4α) or proliferation (proliferating cell nuclear antigen [PCNA]). No significant difference in macrophage marker CD68 (scale bars, 50 μm). (H) Infected armadillo liver (B and G) is phenotypically similar to normal human adult liver. *In situ* expression of the homologous proteins in human livers adapted from Human Protein Atlas (scale shown).

Expressions of specific known liver trophic factors were examined (Figures 3D and 5). FOXA TFs are regulators of embryonic hepatic specification and development, as well as adult biliary function^45^^,^^46^ and parenchymal homeostasis.^47^ The transcriptome from infected animals showed significant upregulation of FOXA1, FOXA2, and FOXA3 (Figures 3D and 5A). We also found that LGR4 and LGR5, promoters of Wnt/β-catenin signaling in development^48^ and involved in control of liver size, growth, and zonation,^5^^,^^49^ are upregulated in ML infection. Further, hepatocyte growth factor (HGF), a potent hepatic mitogen,^50^ and insulin-like growth factors 1 (IGF-1) and 2 (IGF-2), major niche factors for tissue survival, proliferation, and growth expressed in regenerating livers and important stimulants for both mouse and human liver organoid expansion,^5^^,^^51^^,^^52^ are also induced (Figures 3D and S9). In addition, there is induction of other well-described growth factors and their receptors and binding proteins involved in development and regeneration, including BMP1, BMP5, BMP10, TGFB, FGFR4, IGF2R, and IGFBP7^6^^,^^8^^,^^9^^,^^16^ (Figures 3D and S9).

To confirm transcriptional data at the protein level, we used available cross-reactive antibodies (Figures 5B–5G; STAR Methods). We found increased protein expression of developmental or homeodomain TFs FOXA1/2, HNF4α, SOX9, SOX17, and GATA6; adhesion molecules E-Cadherin and ß-Catenin; and trophic factors such as IGF family proteins (Figures 5B, 5E–5G, S4, and S8). Liver functional proteins albumin, HNF4α, AFP, CEBP/α, and CYP members are expressed or upregulated at protein levels from whole-liver extracts and individual cells *in situ* from infected animals (Figures 5B and 5E–5G).

The distribution of hepatocytes expressing FOXA1/2 and HNF4α was examined in detail. HNF4α is upregulated in infected livers and specifically expressed in hepatocytes (Figure S8). Hepatocytes with nuclear FOXA1/2 immunopositivity were pericentral and midzonal but absent from portal and periportal areas (Figure 5B); neither was detectable in uninfected armadillo and normal human liver (Figures 5C and 5D). However, the pattern of expression of other functional liver markers in infected armadillo livers (albumin, E-Catherin, HNF4α, ß-Catenin, and CD68) was similar to normal human liver (Figures 5G and 5H), suggesting ML-infected livers share common molecular and histopathological features with normal human adult livers.

The Hippo pathway is associated with liver growth, regeneration, and cancer development.^53^ Only a few pathway members were differentially modulated in infected livers at gene and protein levels. Moreover, the activation of member proteins by phosphorylation, which is critical for downstream regulation of liver growth,^17^^,^^53^ was absent (Figure S9). Of detectable YAP/Taz target genes, the majority showed no significant difference, and only a few showed marginally increased expression in infected animals (Figure S9D).

### ML influence anti-aging-associated gene patterns in armadillo liver

**Figure 6:**
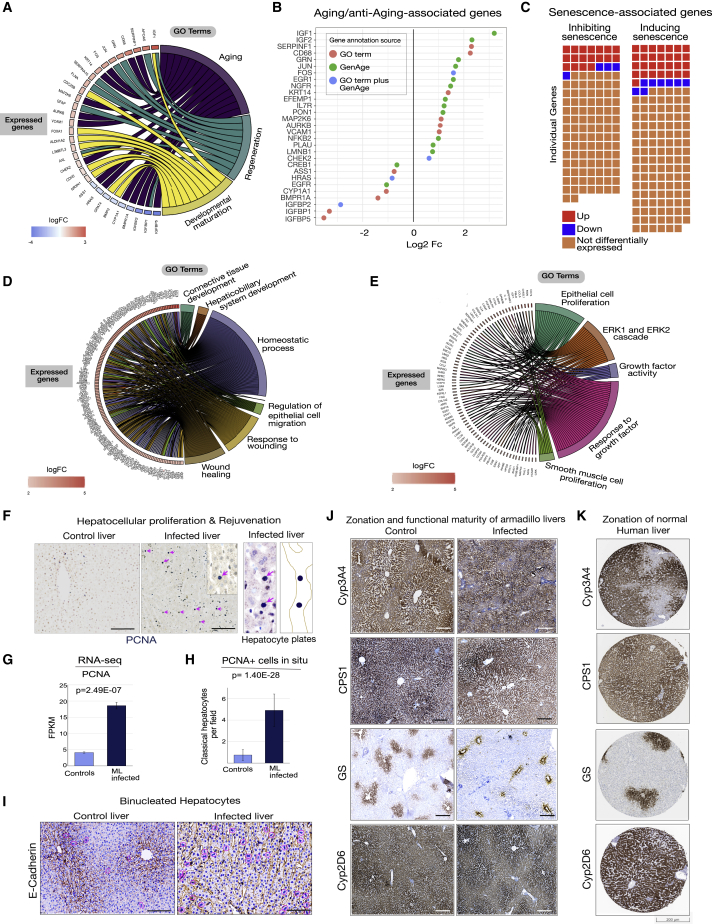
Infected livers undergo dynamic *in vivo* reprogramming, growth and proliferative responses, and activation of regeneration programs while maintaining liver fitness with normal liver zonation and metabolic phenotype (A) Genes upregulated in ML infection with shared mapping to GO terms pertinent to aging, regeneration, and developmental maturation. (B) Individual aging and anti-aging genes differentially expressed in infected livers. (C) The majority of senescence-inducing and senescence-inhibiting genes identified in the CellAge database of cell senescence genes are not differentially expressed in ML-infected liver. (D and E) The GO terms include liver development and homeostasis, wound healing, cell proliferation, and response to growth factors, broadly reflecting tissue growth rejuvenation and regeneration (D), and ERK1/2 cascade, a known ML-induced signaling pathway mediating both rodent and human Schwann cell proliferation,^25^^,^^26^^,^^27^ epithelial cell proliferation, and responses to growth factor (E). (F) PCNA^+^ hepatocytes are increased in infected armadillo livers (right panel and inset; arrows). PCNA^+^ classical hepatocyte nuclei (arrows) with a corresponding schematic demarcating PCNA^+^ cell-containing hepatocyte plates (right images). (G) PCNA is significantly transcriptionally upregulated in infected livers. Data are from three infected and three control armadillos. Scale bars, 50 μm. (H) Quantitative analysis showing increased numbers of PCNA^+^ classical hepatocyte nuclei in infected livers. Presented as mean ± SD from triplicates from three infected and two control armadillos. (I) Increased binucleated hepatocytes in infected livers. A wider distribution of hepatocellular membranous E-cadherin expression demarcates increased number of individual hepatocytes with binucleation labeled with DAPI (circled) in infected liver as compared with control livers. (J) Expression of functional proteins in mature hepatocytes (Cyp3A4, CPS1, glutamine synthetase [GS], and Cyp2D6) shows the same zonal or panlobular expression in infected and control armadillo liver (scale bars, 200 μm). (K) A similar zonal pattern of expression is shown in normal human livers (images from the Human Protein Atlas, scale shown).

The comparison of transcriptomes of infected adult armadillo liver with human fetal liver revealed an inverse relationship with aging-associated genes (Figures 6A and 6B). Genes mapped to the GO term “aging” (specifically IGFBP5, IGFBP1, and IGFBP2) were downregulated in both AFP-positive human progenitor-like hepatocytes and ML-infected armadillo livers, whereas known anti-aging markers such as RGN/senescence Marker Protein-30, which suppresses oxidative stress in the liver and is downregulated during aging,^54^ was significantly upregulated in both infected livers and human fetal livers (Figures 4D and 6A). In addition, because senescence is directly associated with aging,^55^ we also examined senescence-related genes^56^ in infected livers. There was only minimal differential expression of known senescence inducer or inhibitor genes in infected animals, without change in master regulators of senescence, such as p21, p16, and p57. Because senescence is a hallmark of aging, the absence of induction of senescence programs in infected livers aligns with potential bacterial halting of the natural aging process (Figure 6C).

Other liver growth-associated genes map to GO terms, including “regeneration,” “developmental maturation,” “wound healing,” “homeostatic processes,” “response to growth factors,” and “epithelial cell proliferation” (Figures 6A, 6D, and 6E). Importantly, in keeping with our previous demonstration that ML induces proliferation of rodent and human adult Schwann cells using extracellular signal-regulated kinase (ERK) 1/2 signaling pathways,^25^^,^^27^ we mapped ML-induced genes to the GO term “ERK1 and ERK2 cascade” in infected livers (Figure 6E), suggesting a potential common signaling mechanism used by ML for survival, proliferation, and de-differentiation of both Schwann cells and hepatocytes.

### Bacteria-induced hepatocyte proliferation contributes to liver growth

At the transcriptional level, infected armadillos significantly upregulated eight species of cyclins (cyclins A, D, E, G, I, J, and Y) and their cyclin-dependent kinases and also downregulated many cell-cycle-associated genes, suggesting that ML exert a dynamic regulation of cell cycle to balance liver cell proliferation and redifferentiation (Figures 3D, 4H, 4I, 6A, 6D, and 6E). The proliferation marker proliferating cell nuclear antigen (PCNA) was upregulated at transcript level in ML-infected liver (Figure 6F), and there were significantly increased PCNA-immunopositive hepatocytes in infected livers (Figures 6F–6H). Increased binucleated cells and E-cadherin in infected livers may also indicate a regenerative-like hepatocyte phenotype created during ML infection (Figure 6I). Also, in agreement with our previous findings of ML’s capacity to induce proliferation of Schwann cells using ERK1/2 signaling pathways,^25^^,^^27^ we also mapped ML-induced genes to the GO term “ERK1 and ERK2 cascade” in infected livers (Figure 6E), suggesting a potential common signaling mechanism used by ML. Together, these results provide evidence for the contribution of hepatocyte proliferation to bacteria-induced organ growth.

Although we did not find a significant increase of the non-hepatocyte (“other”) cell population that includes immune cells in infected livers (Figure 2), we analyzed the potential involvement of macrophages because they are known to be associated with liver regeneration and disease.^16^^,^^17^ We generated and characterized antibodies specific for nine-banded armadillo macrophages using an amino acid sequence of armadillo CD68 (Figures 5E and S10; STAR Methods). Although anti-CD68 antibodies detect a macrophage population in both control and infected armadillo livers, there was no significant increase in CD68 protein in the total liver lysates or *in situ* CD68^+^ cells in infected livers (Figures 5E and 5G).

### Infected enlarged livers show normal zonation and functional metabolic markers

Infected livers express albumin, bile acid-CoA:amino acid N-acyltransferase (BAAT) (involved in bile acid synthesis), CYP gene family members, and other liver metabolic genes required for normal liver functions (Figures 5A–5C, 6J, and S6). In infected livers, the hepatocellular metabolic functions, including glucose, protein, drug/steroid, and bile acid metabolic processes, are upregulated (Figure S6). *In situ* analysis of liver zonation using mature hepatocyte functional markers (GS, CPS1, Cyp3A4, and Cyp2D6) showed that infected livers not only maintain the pattern of zonation present in uninfected armadillo livers but also demonstrate functional maturity similar to that of normal human liver (Figures 5F, 5G, 6J, and 6K).

Mycobacterial pathogens require host cell provision of lipid for survival, lipid-rich cell wall synthesis, and replication.^57^ In the transcriptome of infected livers, genes mapping to lipid metabolism are greatly upregulated (Figure S6), but no lipid accumulation is evident with a specific lipid staining (Figures S6C and S6D), suggesting bacterial lipid utilization prevents visible accumulation. The liver is at high risk for cancer development when chronic infection and inflammation are sustained.^58^ However, we found no histopathological evidence of dysplasia or neoplasia in infected livers (Figures 1 and 4). To understand this further, we examined known oncogene and tumor-suppressor gene expression and found only minimal differential expression of these in infected animals, corroborating the histological evidence for the lack of tumorigenesis (Figure S7).

## Discussion

Strategies promoting organ growth without abnormality are the goal of regenerative medicine or for rejuvenation during aging. Using an evolutionarily refined *in vivo* bacterial model—*M*. *leprae* and its natural animal host, the nine-banded armadillo—we present a model of adult liver growth without adverse effects during the establishment phase of infection. Our natural model may facilitate the unraveling of *in vivo* endogenous pathways that effectively re-engage liver growth, with potential therapeutic implications for safer liver regeneration and rejuvenation.

Liver disease accounts for 2 million deaths per year.^11^ Although a prime candidate for regenerative therapies, no trial using laboratory-grown stem cells for the treatment of cirrhosis has yielded any licensed therapy.^59^ The failure to develop therapies for solid organ disease using injected stem cells suggests that alternative strategies reflecting organ growth and regeneration complexity should be explored. 2D, 3D, and *in vitro* models have shown advances, but their clinical application to large solid organs is limited.^2^^,^^5^ Moreover, current knowledge of *in vivo* liver regeneration is derived from short-lived rodent injury or hepatectomy models.^8^^,^^16^^,^^50^

Injury or hepatectomy-induced growth ceases when the original liver size is reached, but the mechanism stopping the regenerative response is unknown. The ability to bypass such upper-limit restriction would allow mechanisms of regeneration to be studied without prior injury or cell loss. Our findings show that ML engage endogenous regenerative liver pathways, stimulating organ growth *in vivo* while maintaining intact architecture, vascular systems, and functionality. Although unexpected and unconventional, this evolutionarily refined *in vivo* model may advance our understanding of the native regenerative machinery and determine how it can be engaged *de novo* to permit new organ regrowth strategies for potential clinical use, a conceptual advancement with broader implications in regenerative medicine.

Considering the strictly host-dependent intracellular lifestyle and limited protein-encoding genes,^57^ it is not surprising that ML have evolved sophisticated strategies inducing host cell proliferation, regeneration, and growth.^29^^,^^30^^,^^32^^,^^60^^,^^61^ ML may also take advantage of regenerative and metabolic properties of livers. The metabolically rich liver microenvironment compensates for known ML metabolic defects, inducing numerous metabolic genes in infected livers. Because the observed *in vivo* healthy liver growth is not stimulated by other bacterial species or drug treatment, liver growth appears to be ML specific. Maintenance of an expanded functional liver permits host cell-dependent intracellular bacterial propagation during the establishment phase of infection. Given the variable presence of immune cells but absence of histological cell death or fibrosis, one can speculate that part of the adaptation of the host response by ML involves modulation of innate immune cell activity, preventing tissue damage.

**Figure 7:**
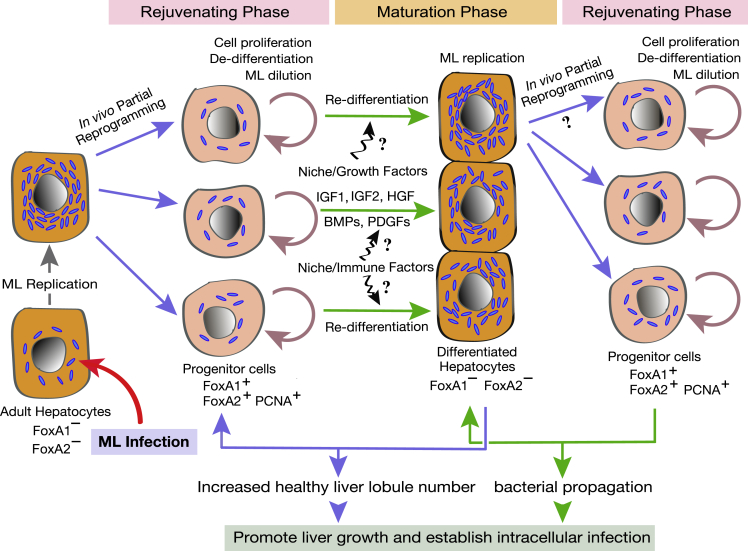
A model for bacteria-induced liver organ growth in living animals Proposed mechanism of cyclical progenitor and mature/differentiated hepatocyte population generation producing liver growth and allowing intracellular bacterial propagation in the adult liver.

We have previously shown that ML hijack the plasticity and regenerative properties of adult Schwann cells by partially reprogramming them into a neural progenitor/stem cell-like state permitting bacterial propagation and dissemination.^28^^,^^29^^,^^30^^,^^32^ Indeed, we showed ML also promote proliferation of adult human Schwann cells,^25^^,^^27^ a finding replicated *in vivo* in sensory neuron-associated armadillo Schwann cells.^62^ Translating this bacteria-induced partial reprogramming to *in vivo* liver organ growth, our data suggest that ML utilizes a similar evolutionarily refined strategy in adult liver by *in vivo* partially reprogramming hepatocytes into liver progenitor-like cells leading to proliferation and subsequent re-differentiation on exposure to niche factors generated within the bacterially created, tissue-regeneration-favorable microenvironment (Figure 7). Indeed, numerous tissue-specific progenitor markers were reactivated or upregulated in infected livers. Heterogeneous *in situ* expression of FoxA1/2, not detectable in uninfected livers, was readily identified in midzonal hepatocytes recently identified as the main source of hepatocellular replenishment in liver homeostasis.^63^^,^^64^ The cyclical, dynamic generation of progenitor and mature/differentiated hepatocyte populations and released trophins potentially leads to this growth (Figure 7). Additional studies are needed to compare the effects of ML infection with a more conventional liver injury-repair approach in armadillos, particularly to identify molecular pathways that discriminate the hepatic response to injury versus ML.

Recent progress in cellular reprogramming has re-visited the potential of OSKM factors in tissue rejuvenation.^19^^,^^20^^,^^21^^,^^22^ In mice, liver-specific expression of OSKM enhanced regeneration by partial reprogramming.^19^ Because long-term overexpression of OSKM *in vivo* leads to the development of totipotent cancers in mice,^65^ the safety of partial reprogramming using OSKM factors that are expressed and activated in many human cancers^66^^,^^67^ in tissue repair or growth must be clarified before clinical consideration. In contrast, in the bacterial model with natural partial liver cell reprogramming, bacteria have evolved protective mechanisms to avoid these adverse effects. Understanding these mechanisms may inform new rejuvenating interventions for both liver and other adult aging organs. Indeed, the findings that ML also downregulate genes associated with aging and upregulate genes involved in the anti-aging process without activating senescence suggest that ML may have evolved to rejuvenate the adult liver, maintaining a “youthful” state with active metabolism favoring host-function-dependent bacterial survival and replication for an extended period. This evolutionarily refined bacterial strategy may also occur in other ML-infected highly regenerative adult tissues, particularly skin and peripheral nerves. Our previous studies have shown partial reprogramming of adult Schwann cells by ML converts infected cells to a “youthful” progenitor/stem cell stage, and on this basis, others have predicted that ML could turn back the clock in host cells.^60^^,^^61^

Importantly, in our model, the entire infected liver grows larger with new tissues *in vivo*, without injury, senescence, fibrosis, or tumor formation, but with normal architecture and function, demonstrating that adult livers can grow *in vivo* without injury or cell loss, and thus regenerative medicine’s pursuit of a “grown-to-order” functional organ is not theoretical but has a naturally occurring precedent. Most critically, understanding how the regenerative machinery can be engaged *de novo* in a long-lived larger mammalian model will potentially allow the development of new and safer organ regrowth strategies for clinical use that could reduce or replace the need for transplantation or rejuvenating aging livers that could facilitate healthy aging.

### Limitations of the study

Our studies are limited by lack of available molecular tools, because the nine-banded armadillo is not a commonly used organism. Considering that much of the known biology comes from short-lived rodent models and their limitations for chronic and aging-associated human diseases and the value of the long-lived and large mammalian models to gain fundamental biologies,^68^ it is worth developing new reagents specific for them. Eventual pathogenic events of late infection have not been addressed. ML cannot be genetically manipulated, but the bacterial genome is a valuable resource for future studies. Future advances that overcome such obstacles could pave the way for our understanding of mechanistic detail as to how ML promote new liver tissue generation and drive liver growth. Aging trajectories and mature cell identity need to be analyzed simultaneously with parameters of known cellular aging hallmarks to further delineate liver rejuvenation *in vivo*, although the endpoint of this natural bacterial approach has already produced “youthful” liver tissues at organ level without adverse effects, which is a safer outcome than one would expect from any experimental regenerative and rejuvenating interventions.

## STAR★Methods

### Key resources table

**Table:** 

REAGENT or RESOURCE	SOURCE	IDENTIFIER
**Antibodies**		

Goat anti-mouse Albumin antibody	Bethyl Laboratories	A90-134A
HNF-4α Antibody (C-19)	Santa Cruz Biotechnology	sc-6556
HNF-4α Antibody (H-171)	Santa Cruz Biotechnology	sc-8987
E-Cadherin (24E10) Rabbit mAb	Cell Signaling Technology	3195
PGL-1 antibody	Gift from Dr. A. Kolk	N/A
Anti-nine-banded armadillo specific CD68 polyclonal antibodies	Rambukkana lab. This paper	N/A
Anti-FOXA2 antibody	Abcam	ab23630
Recombinant Anti-FOXA1 antibody [EPR10881]	Abcam	ab170933
Anti-Sox9 Antibody	Merck/Millipore	AB5535
Human SOX17 Antibody	R&D Systems	AF1924
Human GATA-6 Antibody	R&D Systems	AF1700
β-Catenin (D10A8) XP® Rabbit mAb	Cell-Signaling Technology	8480
Anti-CEBP Alpha antibody [5B7]	Abcam	ab128482
Monoclonal Anti-β-Actin antibody produced in mouse	Sigma-Aldrich	A2228
α-Tubulin (11H10) Rabbit mAb	Cell-Signaling Technology	2125
MST1 Antibody	Cell-Signaling Technology	3682
MST2 Antibody	Cell-Signaling Technology	3952
SAV1 (D6M6X) Rabbit mAb	Cell-Signaling Technology	13301
LATS1 (C66B5) Rabbit mAb	Cell-Signaling Technology	3477
YAP/TAZ (D24E4) Rabbit mAb	Cell-Signaling Technology	8418
IGF1 antibody	GeneTex	GTX100521
Anti-PCNA Antibody, clone PC10 Anti-PCNA Antibody	Millipore Sigma-Aldrich	MAB424R
Anti-CPS1 Antibody	Abcam	ab3682
Anti-Glutamine Synthetase (GS) Antibody	Abcam	ab49873
Anti-CYP3A4 and CYP2D6 antibodies	Gift from Dr. D. Hay	N/A
Goat anti-Rabbit IgG (H + L) Highly Cross-Adsorbed Secondary Antibody, Alexa Fluor 568	Invitrogen/ThermoFisher Scientific	A-11036
Goat anti-Mouse IgG (H + L) Highly Cross-Adsorbed Secondary Antibody, Alexa Fluor 488	Invitrogen/ThermoFisher Scientific	A-11029
Goat anti-Rabbit IgG (H + L) Cross-Adsorbed Secondary Antibody, Alexa Fluor 647	Invitrogen/ThermoFisher Scientific	A-21244
Anti-goat 549 secondary antibody	DyLight	705-505-003
Anti-Goat IgG (H + L), highly cross-adsorbed, CF™ 568 antibody produced in donkey	Sigma-Aldrich	SAB4600074
Anti-mouse IgG, HRP-linked Antibody	Cell-Signaling Technology	7076
Anti-rabbit IgG, HRP-linked Antibody	Cell-Signaling Technology	7074
Donkey anti-Goat IgG (H + L) Secondary Antibody, HRP	ThermoFisher Scientific	A15999

**Bacterial strains**		

*M*. *leprae*: Thai-53, NHDP-63 and NHDP-98 (United States), and BR-4923 (Brazil) strains.	National Hansen’s Disease Program (NHDP), USA	N/A

**Biological samples**		

Human liver tissue	Lothian NRS Human Annotated Bioresource	N/A

**Chemicals, peptides, and recombinant proteins**		

RNAlater	Sigma-Aldrich	R0901

**Deposited data**		

RNAseq data	This paper	Accession code: GSE216223
Whole-slide images of H&E-stained sections of infected, resistant, and control armadillo liver	The University of Edinburgh DataShare repository	https://doi.org/10.7488/ds/3147
O.C.T. Compound	CellPath	KMA-0100-00A
PBS	Oxoid	BR0014G
Sucrose	Sigma-Aldrich	S5016
Goat serum	Life Technologies	10000C
BOND Polymer Refine Detection	Leica Biosystems	DS9800
Oil Red O	Sigma-Aldrich	00625
FluorSave Reagent	Merck/Millipore	345789
T-PER™ Tissue Protein Extraction Reagent	ThermoFisher Scientific	78510
Halt™ Protease Inhibitor Cocktail (100X)	ThermoFisher Scientific	87786
BCA Protein Assay Kit	Boster	AR0146
SeeBlue™ Plus2 Pre-stained Protein Standard	ThermoFisher Scientific	LC5925
NuPAGE™ LDS Sample Buffer	Invitrogen/ThermoFisher Scientific	NP0007
NuPAGE™ 4 to 12%, Bis-Tris, 1.5 mm, Mini Protein Gel, 10-well	Invitrogen/ThermoFisher Scientific	NP0335BOX
NuPAGE™ MOPS SDS Running Buffer (20X)	Invitrogen/ThermoFisher Scientific	NP0001
NuPAGE™ Antioxidant	Invitrogen/ThermoFisher Scientific	NP0005
Immobilon-P PVDF Membrane	Merck/Millipore	IPVH00010
NuPAGE™ Transfer Buffer (20X)	Invitrogen/ThermoFisher Scientific	NP0006
Methanol	Fisher Scientific	M/3900/17
Nonfat Dry Milk	Cell-Signaling Technology	9999
TWEEN® 20	Sigma-Aldrich	P1379
Western Blocker™ Solution	Sigma-Aldrich	W0138
Recombinant armadillo CD68 protein (rCD68)	This paper	N/A
Triton™ X-100	Sigma-Aldrich	X100
Amersham ECL Prime Western Blotting Detection Reagent	GE Healthcare	RPN2232
DAPI (4',6-Diamidino-2-Phenylindole, Dilactate)	Invitrogen/ThermoFisher Scientific	D3571
ProLong™ Diamond Antifade Mountant	Invitrogen/ThermoFisher Scientific	P36965
TRIzol™ Reagent	Invitrogen/ThermoFisher Scientific	15596026
Chloroform Pure	Scientific Laboratory Supplies	CHE1576
2-Propanol	Sigma-Aldrich	I9516

**Experimental models: Organisms/strains**		

Nine-banded Armadillo (*Dasypus novemcinctus*)	National Hansen’s Disease Program (NHDP), USA	N/A
*Mycobacterium leprae* strains: THAI53, Brazil, NHDP63 and NHDP98	National Hansen’s Disease Program (NHDP), USA	N/A

**Software and algorithms**		

Zen software	Zeiss	https://www.zeiss.com/microscopy/int/products/microscope-software/zen-lite.html
Image Studio Lite	LI-COR	https://www.licor.com/bio/image-studio-lite/download
Image J/Fiji	^69^^,^^70^	https://imagej.net/Fiji
GraphPad Prism (v8)	GraphPad	https://www.graphpad.com/scientific-software/prism/
RStudio (R 3.3.3)	R Core Team, 2016	https://www.rstudio.com/
TissueStudio 2.4 (Definiens AG, Munich, Germany)	Definiens	N/A
DeveloperXD 2.7 (Definiens AG, Munich, Germany	Definiens	N/A
Solexa pipeline v1.8 (Off-Line Base Caller software, v1.8)		N/A
FastQC software (v0.11.7)	^71^	N/A
cutadapt (v1.17)	^72^	N/A
Hisat2 software (v2.1.0)	^73^	N/A
StringTie (v1.3.3)	^74^^,^^75^	N/A
Ballgown (v2.10.0)	^76^^,^^77^^,^^78^	N/A
CPAT (v1.2.4)	^79^	N/A
GSEA	Broad Institute, MA	https://www.gsea-msigdb.org/gsea/index.jsp
REVIGO	^80^	N/A
GOplot	^81^	N/A
*In situ* expression of proteins from normal human liver presented in this study is adapted from the Human Protein Atlas	The Human Protein Atlas Project:	https://v15.proteinatlas.org/about/project^82^

### Resource availability

#### Lead contact

Further information and requests for resources and reagents should be directed to and will be fulfilled by the lead contact, Anura Rambukkana (a.rambuka@ed.ac.uk).

#### Materials availability

Enquiries regarding this study should be directed to the lead contact. All reagents generated in this study are available from the lead contact without restriction.

### Experimental model and subject details

#### Nine-banded armadillo acquisition and preparation

*In vivo* armadillo studies were undertaken at the United States Department of Health and Human Services, Health Resources and Services Administration, Healthcare Systems Bureau, National Hansen’s Disease Program (NHDP), Baton Rouge, Louisiana, USA. Both captive-born (provided by Frank Knight, University of Ozarks, Clarksville, Arkansas) and locally wild caught armadillos were used. In total, 57 armadillos were used in this study, comprising 12 uninfected controls, 13 infected resistant and 32 disseminated animals (Suppl data Table S1). Of these all disseminated, 8 uninfected controls and 9 resistant armadillos were used for liver: body weight analysis presented in Figures 1A–1C and S1. In addition, 4 uninfected/controls and 4 resistant animals were used in histological and DAPI screening for machine-learning/computational analyses.

#### Wild armadillo husbandry

Free ranging armadillos were taken by local trappers and transported to the National Hansens’s Disease Program (NHDP) vivarium where they were housed in modified rabbit cages and conditioned for experimental inoculation with viable *M*. *leprae*. During conditioning the animals were treated for intercurrent bacterial and parasitic infections and adapted to the laboratory environment as follows: 1) the formulary used in armadillo maintenance is highly restricted to 1 antibiotics (ampicillin), 1 antihelminth (ivermectin), and 1 injectable (Detomidine with Xylazine) and 1 gas anaesthesia (isolflourane). None have consequential liver effects. Animals were maintained in captivity for more than 1 year before being infected and all of the non-infected animals were exposed to these same agents as the infected. Thus, the non-infected tissues are appropriate controls for these and other unknown environmental variables.

To test if armadillos harboured a pre-existing infection with *M*. *leprae*, animals were held for 3–6 months and tested twice for serum antibodies to *M*. *leprae*-specific phenolic glycolipid-1 (PGL-1). After conditioning, the animals were skin tested using Lepromin (a suspension containing 10^7^ killed bacilli) to determine the type of granuloma they may form in response to *M*. *leprae*, and lepromatous/multibacillary animals (armadillos that extensively propagate bacilli in tissues) likely to develop disseminated disease were selected.

#### Captive-born animal husbandry

Gravid females were caught in the wild and kept in captivity where they gave birth to four genetically identical clonal siblings. The young were brought to the NHDP vivarium at approximately 4 months of age and conditioned for a longer period (up to 2 years depending on size and weight gain) than the wild caught armadillos. During conditioning they were routinely dewormed, treated with antibiotics as described above for intercurrent issues, lepromin tested, and screened for anti-PGL1 antibodies for evidence of any prior *M*. *leprae* infection.

#### Ethical approval

The study was approved and conducted within the ethical guidelines outlined under the U.S. Department of Agriculture Animal and Plant Health Inspection Service and the U.S. Public Health Service Policy for the Care and Use of Laboratory Animals (NHDP IACUC assurance number A3032-01). This approval is a part of the interagency agreement by the National Institutes of Health, National Institute of Allergy and Infectious Diseases for providing *in vivo* grown leprosy bacilli and other leprosy research reagents to researchers worldwide.^83^

#### Comparative studies on human liver tissue

Human tissue was obtained by application to the Lothian NRS Human Annotated Bioresource under ethical approval number 15/ES/0094 from East of Scotland Research Ethics Service REC 1 that authorizes anonymized unconsented access to tissue. Formalin-fixed paraffin-embedded sections of liver with the stated chronic liver diseases, or histologically normal liver in resections for metastatic malignancy, were H&E and PSR stained for illustrative purposes. Anonymized tissue sections were provided by the Bioresource with a diagnosis only (indicated in figure legends), and patient age and gender are not known to the authors.

### Method details

#### Selection of armadillos for disseminated infection by lepromin test

The Lepromin test determines the histopathological response to *M*. *leprae* using lepromin, which consists of a suspension of whole, autoclaved *M*. *leprae* derived from athymic nude mice footpads^36^^,^^84^ containing phenol as preservative. Lepromin was inoculated intradermally in the abdomen and the area was tattooed for later identification. Three weeks after injection a skin sample was collected using a 6 mm biopsy punch, and the biopsy was fixed in neutral buffered formalin for further processing and Wafe-Fite staining to detect acid-fast bacilli. A negative response to the test is associated with the multibacillary form of leprosy and an inability to mount a T-cell-mediated immune (CMI) response to *M*. *leprae* (similar to human lepromatous/multibacillary leprosy form) whereas a positive response is indicative of a high CMI-associated granulomatous response and successful elimination of the bacilli (resembling human tuberculoid/paucibacillary leprosy). The majority of the Lepromin test negative armadillos progress to a fully disseminated infection while animals that manifest a Lepromin positive response develop only mild disease. Therefore, only armadillos that did not respond to lepromin, likely to propagate *M*. *leprae* infection, were used for final inoculation with viable *M*. *leprae*.

#### Experimental inoculation with viable *M*. *leprae*

The *M*. *leprae* preparation was freshly harvested (<48 h) from athymic nude mouse footpads, and viability and contamination with other bacteria were determined as previously described.^36^^,^^84^ We used strains of *M*. *leprae* from 3 different geographical origins: Thai-53 (Japan), NHDP-63 and NHDP-98 (United States), and BR-4923 (Brazil). These strains exhibit minor genotypic variability but have no known pathological variation among humans.^85^ There was no difference in bacterial propagation or liver growth (liver weight: body weight ratio) from infected animals with ML strains. The average age at inoculation was 24 months and the average body weight was 8–10 pounds (4–5 kg). Underweight animals or those demonstrating any other abnormality were excluded from study. Armadillos were anesthetized using a combination of Ketamine (0.6 mL) and Dexdomitor (0.4 mL) given intramuscularly after the skin at the injection site was cleaned with alcohol. The inoculum, which consisted of 1 × 10^9^ viable *M*. *leprae*, was injected slowly in the saphenous vein in a maximum volume of 0.5 mL.

#### Monitoring infection progression

After inoculation, animals were screened serologically by ELISA every three months for PGL-1 and LID1 antibodies (Suppl data Figure S1). When a positive ELISA assay (Optical Density (OD) > 0.700 measured at 540 nm) was detected, the animals were followed on a monthly basis for progression of infection, liver functions and dissemination by serology and blood serum levels of liver function enzymes (Figure 5H). On average, the infected animals show anti-PGL-1 antibodies at 9 months after *M*. *leprae* inoculation. By 18–24 months post-inoculation, most animals develop a severe infection with up to 10^12^ recoverable bacilli from a single armadillo (Figure 1 and Suppl data Figure 1). The armadillos are usually sacrificed when generalized dissemination of bacilli is reached and the animals show highly positive PGL-1 ELISA. At the time of sacrifice, disseminated animals showed no apparent physical abnormalities. The majority of lepromin-negative armadillos are susceptible to infection with viable *M*. *leprae*. However, 20% of the animals will resist challenge. The animals are considered resistant if they do not show serum anti-PGL1 antibodies above the cut-off value of 0.700 OD or signs of dissemination after 40 months post-inoculation (Suppl data Figure S1C). At sacrifice, these animals will show a considerably lower bacterial count in the liver tissue compared to those that disseminate (Figures 1B and S1C).

#### Ultrasound imaging of liver of live armadillos during infection

The liver images of live normal, resistant and disseminated *M*. *leprae*-infected armadillos were generated using an ultrasound composed by a 15 MHz transducer probe connected to a scanner (MicroUS) which in turn is connected to the summit base unit (Cadwell, USA). The animal was anesthetized, placed in a supine position, and the transducer was positioned on the right side of the abdomen directed cranially towards the thorax. The depth was set to 70 mm to compare the size of the liver to other organs and to 50 mm to measure the area of the liver for comparison among animals (Figure 1D).

#### Sacrifice and harvest of tissues

Before sacrifice, animals were given Gentamicin and Penicillin as a precaution against low level secondary bacterial contamination. The animals were anesthetized, shaved, and thoroughly cleaned with iodine, alcohol, and sterile water. After euthanization the liver was removed, placed in a sterile jar and weighed before being placed on ice (Suppl data Figure S1A). Tissue samples were collected and fixed in neutral buffered formalin and RNAlater for histological examination and molecular analyses. Tissues were transported to the laboratory for sterility testing and AFB enumeration and stored at −80°C until use for molecular analyses.

#### Tinctorial staining

Formaldehyde-fixed armadillo liver tissue was embedded in paraffin blocks, or frozen in O.C.T. compound following PBS washes and overnight 30% sucrose in PBS solution incubation. Sections were cut onto glass slides, using a microtome or cryostat. For Haematoxylin and Eosin (H&E) staining of armadillo liver, 10 μm paraffin sections were dewaxed and rehydrated, before incubation in ready-made Harris Haematoxylin. Slides were washed in running water, then differentiated for no more than 5 s in 1% acid alcohol, before transferring to Scott’s tap water substitute. Some sections from frozen armadillo liver were also used in this study. Slides were then stained in eosin solution, washed in running water, dehydrated, cleared in xylene, and then mounted with Pertex and glass coverslips by hand or with Shandon Thermo Scientific™ ClearVue™ Coverslipper. For Picro Sirius Red (PSR) staining, 10 μm paraffin sections on glass slides were dewaxed and rehydrated before placing into PSR solution for 2–4 h. Slides were then rinsed in 100% IMS, cleared in xylene, and mounted.

#### Digital slide acquisition and expert histopathological screening

For illustrative figures, images were captured on Zeiss Observer microscope with 20x objective lens capturing tiles regions with Zen software. Whole-slide images of H&E-stained sections were captured on a Hamamatsu NanoZoomer to ×20 depth; all whole-slide images are available from University of Edinburgh DataShare repository - https://doi.org/10.7488/ds/3147. All H&E and PSR histologically stained sections were examined by a Consultant Liver Histopathologist (TJK) working at the national liver transplant center, blinded to infection status, and all photomicrographs reproduced in the manuscript were reviewed and quality-assured by TJK. Gold-standard subjective evaluation/slide reporting as routinely undertaken on standard human clinical specimens was provided.

#### Antibodies, immunolabeling and confocal microscopy

Most of the immunolabeling labelling procedures were adapted from our previous lab protocols (Masaki et al., 2013; Ng et al., 2000). Frozen 10 μm Armadillo liver sections were methanol fixed at −20°C for 15 min, washed with PBS, and then blocked with 10% Goat serum or Horse serum in PBS for 1 h at room temperature. After blocking, sections were incubated with primary antibodies diluted in blocking solution overnight at 4°C. It should be noted that a large number of commercially available antibodies to mouse and human markers were tested in armadillos but only the small number of antibodies that reacted are documented below; none of the antibodies to immune markers showed any activity.

#### Primary antibodies for immunofluorescence

We used the following antibodies: Albumin (1:100), HNF4α (1:50–1:100), E-cadherin (1:100), PGL-1 (Ng et al., 2000) (1:500). Sections were washed in PBS and incubated with secondary antibodies diluted in blocking solution for 90 min at room temperature. Secondary antibodies include: Invitrogen AlexaFlour; anti-rabbit 568, anti-mouse 488, anti-rabbit 647, DyLight; anti-goat 549, Sigma; anti-goat 568. Sections were stained with DAPI dilactate, washed with PBS and dH_2_O, and mounted under glass coverslips using Fluorsave mounting media. For the detection of macrophages in the liver we generated armadillo specific CD68 antibodies (see below).

#### Nine-banded armadillo-specific anti-CD68 antibody generation

None of the commercially available antibodies to mouse and human immune markers were reactive with armadillo immune cells. Since macrophages are resident in the liver and are known to play a role during infection and also liver regeneration, we generated polyclonal antibodies specifically directed to a nine-banded armadillo sequence of macrophage marker CD68 which shared with several macrophage subtypes and Kupffer cells, using the services of ProteoGenix (Suppl data Figure S12). The amino acid sequence of the extracellular domain of the nine-banded armadillo with added His-tag, shown below, was used to generate a recombinant armadillo CD68 protein (rCD68) for immunizing rabbits. Armadillo CD68 amino acid sequence used for generating rCD68 is shown below:

MGKDCPHKKSATLLPSFTVTPTATESTASTATASHRTTKSHKTTSHKTTTHRTTTHQPTTHQSTTSPGPTNATHNPATTTSHGNATVHPTSNSTTSQGTTSTSSPHPRPPPPSPSPSPGSKEAEGDYTWLNGSQPCIRLQAQIQIRVLYPTQDGEEAWGISVLNPNKTKAEGECGGAHAHLLLTFPYGQLSFGFKQEPTQGTVYLNYMAMEYNVSFPRTTQWTFLAENASLGDLQAPLGRSFSCRNASIMLSPALHVDLLSLQVQAAQLPPTGVFGPSFSCPSDQGSHHHHHH.

This rCD68 was expressed in *E*. *coli*, purified from an affinity column and checked for purity by Coomassie blue staining after protein separation by gel electrophoresis. Purity was confirmed as a single band corresponding approximately to a 35 or 37kDa protein. Using the rCD68 as immunogen polyclonal antibodies were raised in two rabbits, with monitoring of serum antibody reactivity to rCD68 by ELISA with OD450nm. These anti-CD68 antibodies were then affinity purified and tested for specific reactivity against 0.5μg rCD68 protein using two antibody dilutions (1:8,000 and 1:16,000) by western blot validation. Subsequently, anti-CD68 antibodies were determined to react specifically in armadillo liver protein extracts from control, 19-, 24- and 30-month infected animals across a range of loading concentrations (20, 50 and 100μg) in western blots, with clear, single bands observed. Specific detection of macrophages was also observed in immunofluorescence in Armadillo livers, as compared to antibody controls (Suppl data Figure S10; also shown in the main Figures 5A and 5C).

#### Immunofluorescence

For some antibodies, antigen retrieval was performed in citrate buffer pH6. Sections were washed sequentially in PBS, PBS 0.1% Triton X-100, PBS 0.01% Triton X-100, PBS, at 4°C, before blocking in 5% goat serum in PBS for 1 h at room temperature. Sections were incubated in primary antibodies diluted in PBS 0.01% Triton X-100 1% goat serum at 4C overnight. After PBS washes, sections were incubated in secondary antibodies diluted in PBS 0.01% Triton X-100 1% goat serum for 1 h at room temperature, before DAPI dilactate staining, washing and mounting. Images were captured on a Nikon Eclipse 2100 epifluorescence microscope with 20x objective, an inverted widefield Zeiss Observer microscope with 20x objective, and a Zeiss LSM710 confocal microscope with 40x or 63x objective running Zen software.

#### Immunohistochemistry

IHC was carried out by the professional histology services of SuRF (QMRI, Edinburgh) on 10 μm frozen Armadillo liver sections. All samples were peroxide blocked followed by primary antibody incubations for 1 h at room temperature. Primary antibodies used: FOXA1 (1:100), FOXA2 (1:200), PCNA (1:20), CYP2D6, CYP3A4 (1:400), CPS1 (1:1000), GS (1:5000), E-cadherin (1:100). Irrelevant antibodies or primary antibodies that did not react with armadillo tissues were used as negative controls. Primary antibodies were followed by polymer and DAB incubations, hematoxylin counterstain, and mounting with cover glass. All steps included TBST washes in between, and the procedure utilized Bond polymer refine detection. Images were captured on an inverted widefield Zeiss Observer microscope with 20x objective lens and tile scanning, running Zen software, or a Zeiss Axioscan slide scanner microscope.

#### Oil red O staining

Oil red O (ORO) staining was performed on frozen 10 μm Armadillo liver sections, according to published methods.^86^ As a positive control, sections of steatotic human liver were used. Slides were left to equilibrate for 10 min at room temperature, covered with filtered ORO working solution for 5 min, before washing for 30 min with running water. Slides were mounted with Fluorsave and glass coverslips and sealed with nail polish prior to imaging on an Olympus BX61 upright widefield microscope with a 10x objective lens.

#### Protein extraction and western blots

Protein lysates were collected from freshly frozen armadillo liver tissue by homogenizing 1 g tissue per 20 mL T-PER reagent containing Halt protease inhibitor cocktail. Samples were clarified at 10,000g for 5 min to the remove tissue debris, and supernatant stored at −80°C until used. Total protein was quantified using BCA assay. Western blotting was performed using our previous described protocol (22, 38, 52, 53). Protein lysates and standards were run at appropriate concentration (100μg/lane) with NuPAGE sample buffer, after heat treatment at 95°C for 5 min to denature. Proteins were run on pre-cast NuPAGE 4–12% Bis/Tris Gels at 200V in 1x NuPAGE MOPS SDS running buffer containing 0.5 mL NuPAGE antioxidant. Proteins were transferred to a membrane in a NuPAGE blotting system and 1x Transfer buffer containing 10–20% methanol, for two hours at 20V. The membrane was then blocked in 5% non-fat milk powder in PBS-T (PBS containing 0.5% Tween-20), or in BSA in PBS for 1 h at room temperature on a rocking platform. Blocking solution was removed, and primary antibodies added diluted in blocking solution or Western blocking reagent.

Primary antibodies used in Western blotting include the following: Albumin (1:250), HNF4α (1:100), FOXA2 (1:200), SOX9 (1:100), SOX17 (1:500), GATA6 (1:25), E-cadherin (1:500), β-catenin (1:2000), CEBP/α (1:100), CD68 (see previous section, 1:100), β-actin (1:3000), α-tubulin (1:3000), MST1 (1:300), MST2 (1:300), SAV1 (1:300), LATS1 (1:300), YAP/TAZ (1:300), IGF1 (1:500). Primary antibody incubation was overnight at 4°C on a benchtop rotator. Following this, the membranes were washed three times for at least 10 min each in PBS-T, on a rocking platform. Secondary antibodies, diluted in blocking solution, were added and incubated for 1 h at room temperature with rocking. Secondary antibodies include: Cell Signaling Technology; anti-mouse HRP (1:3000), anti-rabbit HRP (1:3000), ThermoFisher; anti-goat HRP (1:3000). Membranes were washed three times, 10 min each, in PBS-T and then exposed to ECL Prime detection reagents as per manufacturer’s instructions. Protein bands were detected using a LI-COR Odyssey Imager using Image Studio software, and bands quantified using Image Studio Lite and Image J.

#### Analysis of liver functional enzymes in blood samples

Blood serum levels of AST, ALT and LDH were measured using Element DC Veterinary Chemistry. Analyzer, which is a diagnostic medical device that analyses blood by calorimetric assay using DRI-CHEM slides. Analyses were performed according to the manufactures instructions (HESKA). Graphing and statistical analysis of the data was carried out using GraphPad Prism (v8) software.

#### Hepatic lobular architectural analysis of adult armadillo livers

Whole-slide images of H&E-stained sections were acquired using a Hamamatsu NanoZoomer to ×20 depth and exported as.ndpi files. Tiled-TIFF thumbnails were generated from the.ndpi files using ndpisplit from the NDPITools suite,^87^ and tiled-TIFF files converted to jpeg by command-line ImageMagick for annotation using the FIJI implementation of ImageJ.^69^^,^^70^ The centre of each central vein and centre of each hepatic artery (identifying portal tracts when paired with a portal vein branch or bile duct) were separately annotated by the pathologist. Analysis of the relative positions of the vascular structures was undertaken in RStudio (R 3.3.2).^88^ For each image, vascular positions were imported using the RImageJROI package read.ijroi() function,^89^ and converted into spatstat package format using the ij2spatstat() function.^90^ The distances from each central vein to the 6 nearest portal tracts were calculated with the nndist function. To estimate individual liver lobule size, based on the classical lobule depiction as a regular hexagon, the mean of the distances from each central vein to the nearest three portal tracts (r) was used to calculate the area:

$$\left(\frac{3\sqrt{3}}{2}\right).r^{2}$$

The distribution of the vascular structures was determined using Ripley’s L-function, as previously described.^91^

#### Tissue staining and processing for hepatic cellular composition analysis

Cellular composition of the armadillo livers was assessed by taking advantage of characteristic hepatocyte nuclear appearances by DAPI nuclear staining and machine learning identification of hepatocytes and non-hepatocytes or other cells. For this analysis, armadillo liver paraffin sections of equal thickness (4 μm) were de-waxed and rehydrated, then incubated in DAPI dilactate diluted in dH_2_O, washed in distilled water, and mounted in prolong diamond antifade mountant. All liver samples were processed together at the same time to minimize variability. DAPI-stained liver tissue sections were imaged on a Zeiss Axioscan.Z1 (Carl Zeiss AG, Oberkochen, Germany) at 20x using fluorescence filters configured for DAPI and FITC (to acquire autofluorescence). Whole slide.czi files were imported into TissueStudio 2.4 (Definiens AG, Munich, Germany) for automated tissue detection and nuclear segmentation using Tissue Studio’s built-in nuclear detection function. TissueStudio workspaces were then opened in DeveloperXD 2.7 (Definiens AG, Munich, Germany) where the following processing and analysis was conducted: Six 500 X 500pixel regions (1 ROI taken from each of two tissues selected from each animal group) were manually classified for nuclear type by an expert liver pathologist (TJK).

#### Machine learning algorithm for cell classification: Generation and application of a decision tree model for quantitative cellular composition analysis

The six classified ground truth, gold-standard, pathologist-scored ROIs were then used to train a decision tree model (7 depth, 1 sample per leaf, 5-fold cross validation) to distinguish between single hepatocyte nuclei and non-hepatocytes (details of model parameters and feature set can be found in Table S2A). Seventy percent of the pathologist-classified hepatocytes and 70% of the non-hepatocytes were used to train the models (training population), with the remaining 30% of each population used to test the accuracy of the model (test population). Using the features defined in Table S2A, we were unable to reliably classify binuclear hepatocytes as part of a decision tree model because of the morphological similarities between dense touching non-hepatocyte nuclei and binuclear hepatocyte nuclei and so a decision was taken to first use machine learning to classify hepatocytes vs non-hepatocytes and then to reclassify binuclear cells in a stepwise fashion. Therefore, following classification of cell objects in the training ROIs into hepatocytes and non-hepatocytes, a series of stepwise automated class reassignments based on morphology and object context were then used to reclassify those nuclei to be considered binuclear (see stepwise binuclear reclassification pipeline Table S2B). Machine-learning-classified cells and their matching manually classified “ground truth” cells in the test population were then compared to generate an error matrix and calculate statistics for each iteration of the decision tree model. The optimal model parameters were determined by choosing the model that most equally balanced the error matrix statistics between training and test populations. The error matrix for the optimal model can be found in Table S2C. Compared to the test population, the optimum decision tree model achieved positive and negative predictive values of 0.92 and 0.88, respectively, with sensitivity, specificity, and accuracy of 0.93, 0.88, and 0.91 and we considered these values to be acceptable for whole-tissue quantification. Rule-based stepwise reclassification of hepatocyte nuclei to binuclear, by definition, required no model to be trained and thus there was no set training population. For this reason, accuracy of the reclassification was tested on the entire manually classified ground truth binuclear and non-binuclear populations giving PPV 0.84 and NPV of 0.99 (Table S2C). After manually removing artefactual ROIs from each sample’s whole-slide image, the optimal decision tree model and subsequent binuclear-classification pipeline was applied to all full-size, whole-slide images of liver sections by first detecting the nuclei in Tissue Studio and then applying the processing and machine learning pipeline in Developer XD.

#### Detailed machine-learning stepwise workflow for armadillo liver cell classification

Armadillo tissue sections were stained with DAPI and imaged on AxioScan.Z1 whole slide scanner. CZI image files imported into Definiens TissueStudio 2.4 Immunofluorescence Portal. Whole tissue detection (auto-threshold, single tissue options applied) and nuclear detection using TissueStudio’s built-in nuclear segmentation tools operating on the DAPI channel (nucleus region = 1, Typical Nucleus Size = 70um^2^). The whole batch was then processed together with the same tissue and nuclear detection settings (Sol_DetectNuclei_TS.dax). The resulting TissueStudio Workspace was saved and opened in Definiens DeveloperXD 2.4. One candidate region was selected from each of six images covering the range of tissue and nuclear morphology. Training regions were cropped along with nuclear objects then exported and sent to pathologist. Pathologist manually classified all cells in every region and created coloured masks of nuclear classes using Tissue-Studio-segmented nuclear objects as a mask (ImageJ) (Figure 3A). Coloured class masks were imported into Definiens DeveloperXD workspace. (Sol_createTraining DataAndTrainModels.dcp). Training regions were arranged and fused into a single montage with unclassified nuclear objects intact. (Sol_createTrainingDataAndTrainModels.dcp – execute first do loop sections 1-8). Nuclei in training montage were then classified in DeveloperXD by linking mask colour from pathologist classifications to nuclear classes (Hepatocyte, Binuclear, non-Hepatocyte) to create a Definiens-compatible pathologist-trained set of nuclear objects. (Sol_createTrainingDataAndTrainModels.dcp – execute first do loop sections 9&10) 30% of all pathologist-classed nuclei (537 hepatocyte nuclei, 340 non-hepatocyte nuclei) were classified as a test/validation object and not used in training the model. All training and test objects were copied to a new map onto which the model would be applied (“Test Map”). The following section was repeatedly cycled for iterative training using the Definiens Customer Process “Sol_createTrainingDataAndTrainModels.dcp” (section “train and apply decision tree for morphological and intensity classification max/min”).

1 - A decision tree model using the features and metrics described in the materials and methods was trained using the training set of nuclear objects. (Open the configuration window for command “[on elongated, hepatocyteNuc: Classifier: Train…]” in the “train and apply decision tree for morphological and intensity classification max/min” section then set modifiable parameters: Tree Depth, Samples Per Leaf, Cross-fold validation before executing the command)

2 – Nuclear objects on Test Map were converted to a temporary class in preparation for model application (execute command “binuclear, elongated… on NuclearLevel: tempNuc” – resets all nuclear classes to temporary class).

3- The trained model was applied to nuclear objects in Test Map of the nuclear training montage (tempNuc objects) (execute command “tempNuc at NucleusLevel: Classifier: Apply”).

4 - error matrix was calculated by comparing each pathologist-classed nucleus object to its matching computer-classed objects in “Test Map” and calculating relevant statistics (execute command: “Compute Stats for Hepatocyte Vs Elongated only”).

5 – one modifiable parameter (either depth, or samples per leaf, or cross fold validation) in the decision tree model configuration window was then adjusted incrementally.

6 - model was retrained by going back to step 1

Training was iterated and repeated until the error matrix from the internally cross-validated training set model resulted in values that were balanced when the model was applied to the test data set. That is, the error matrices in both training data set and test data set are as comparable as possible given the possible iterations of configuration parameters. Visual confirmation of results is also agreed by pathologist.

#### Optimum model error matrix of model applied to training data set and to test/validation data set

**Table:** 

	PPV	NPV	Sensitivity	Specificity	Accuracy	falsePos/RealPos	FDR	FNR	FOR
trained cells	0.9668	0.9165	0.9404	0.953	0.9455	0.0323	0.0332	0.0596	0.0835
test cells	0.9239	0.8846	0.9274	0.8794	0.9088	0.0763	0.0761	0.0726	0.1154

Training sample set: 1209 hepatocytes, 829 non-hepatocytes. Validation Set: 537 hepatocytes, 340 non-hepatocytes. Decision tree model was then applied across all tissues onto the nuclei segmented using TissueStudio. (execute Sol_ApplyModelsAndExport.dcp as a batch process)

#### RNA extraction from armadillo livers

Liver samples previously freshly isolated and stored frozen in RNAlater were thawed prior to use and then homogenized in TRIzol at room temperature. The RNA fraction was collected using chloroform and isopropanol-based extraction. Total RNA was resuspended in distilled, RNase-free water and quantified using a Nanodrop ND-1000 Spectrophotometer.

#### RNA-sequencing of livers from control and infected armadillos

Triplicate RNA samples isolated from 24-month ML-infected, 30-month ML-infected and two control livers were submitted to Arraystar Inc. (Rockville, USA) services for paired-end RNA-sequencing. 1–2 μg total RNA was used to prepare the sequencing libraries. Library preparation involved oligo (dT) magnetic bead mRNA enrichment, highly strand-specific dUTP method using KAPA Stranded RNA-Seq Library Prep Kit, library size distribution and yield QC with Agilent 2100 Bioanalyzer and by absolute quantification qPCR. To sequence the libraries on the IlluminaNovaSeq 6000 instrument, the barcoded libraries were mixed, denatured to single stranded DNA in NaOH, captured on Illumina flow cell, amplified *in situ*, and subsequently sequenced for 150 cycles for both ends. Image analysis and base calling were performed using Solexa pipeline v1.8 (Off-Line Base Caller software, v1.8). Sequence quality was examined using the FastQC^71^ software (v0.11.7).

For sequencing quality control, raw data files in FASTQ format were generated from the Illumina sequencer and the sequencing quality examined by plotting the quality score for each sample. Quality score Q is logarithmically related to the base-calling error probability (P): Q = −10log10(P). Q30 means the incorrect base calling probability to be 0.001 or 99.9% base calling accuracy, and high-quality data was indicated by the percentage of the number of bases with Q ≥ 30 being greater than 80%. The trimmed reads (trimmed 5′, 3′-adaptor bases using cutadapt^72^ (v1.17)) were aligned to the reference genome (*Dasypus novemcinctus*, Dasnov3.0, GCA_000208655.2 Ensembl) using Hisat2 software (v2.1.0)^82^. The Ensembl Dasnov3.0, INSDC Assembly GCA_000208655.2 was last updated/patched in May 2016, which is well annotated with 22,711 coding genes. The transcript abundances in FPKM at gene and transcript levels were assembled and computed with StringTie (v1.3.3)^83,84^. The differential gene expression was analysed using R package Ballgown (v2.10.0)^85–87^. The novel genes and transcripts not present in the reference genome/transcriptome were predicted by StringTie and their protein coding potentials were scored by CPAT (v1.2.4)^88^.

#### Bioinformatics and differential expression visualization

Sets of total protein coding genes from human (GRCh38.p13) and armadillo (Dasnov3.0) reference genomes were acquired from Ensembl Biomart, and subsequently filtered for genes annotated with gene symbols for cross-comparison. Total armadillo protein coding genes with and without gene symbol annotations were queried against all detected genes from RNAseq from any sample and presented as a waffle diagram. Heatmaps of data were produced from sets of differentially expressed genes using pheatmap in R, Python or shell environment (Python and Shell: in-house scripts, Arraystar). For heatmap generation, the log2 transformed FPKM values of the expressed genes were tested by ANOVA across the samples for significant difference in expression (p <= 0.05) and selected for unsupervised hierarchical clustering using Euclidean distance measure and the ‘average’ agglomeration method. The heatmap was scaled row-wise, with the colour scale representing the Z-scores.

Gene ontology analysis was performed using GSEA (Broad Institute, MA) software with differentially expressed genes sets pre-ranked in order of fold change, with minimum gene set sizes set to 10. REVIGO^80^ was used to visualize GO terms in bubble diagrams, and chord diagrams of differentially expressed genes related to specific GO annotations were created using GOplot.^81^ Selection of functional genes of interest used manual literature searches (PubMed) and gene annotations (GeneCards, NCBI). For the liver cell type gene expression pattern analysis, data sets from^39^ were used, specifically the gene expression signatures associated with the distinct clusters identified in that study. Clusters and their gene lists were pooled together into the groups; “hepatocytes”, “cholangiocytes/biliary/EPCAM+”, “Endothelial/Stellate/MyoFB”, “Kupffer” and “NK, NKT and T cell” where possible. These lists were then cross-referenced with the Armadillo liver differentially expressed genes of 30-month infected liver vs. control livers to provide the differentially expressed infected armadillo liver genes potentially associating with particular liver cell types. The top differentially expressed genes in each of these groups, and selected other functional genes of interest, were used to produce heatmaps (software as described above). Common oncogenes and tumour-suppressors were compiled from an independently determined reference list of genes (https://www.arraystar.com/lncpath-cancer-microarrays/) and compared with detected genes in Armadillo liver RNAseq to determine whether those detected were significantly differentially expressed in 24- and 30-month infected Armadillo livers, presented as heatmaps after hierarchical clustering. Differential expression of genes in chronically infected armadillos was compared with the differential expression of genes from a discrete lineage of scar-orchestrating cells in a murine model of fibrogenesis.^43^ The common set of differentially genes overexpressed in infected armadillo liver and in scar-orchestrating cells after profibrotic injury was used for GO term analysis using g:profiler(ref) and visualized using REVIGO and with chord diagrams from GOplot(ref). The specific expression of collagen species was examined. Genes differentially-expressed in 30-month infected liver versus uninfected control liver were compared with published gene expression data from putative adult and fetal hepatic progenitors,^39^^,^^44^^,^^40^ or with gene expression data from a rat partial hepatectomy model,^41^ and the common set up genes used for GO term analysis and visualization, as above.

To identify changes in senescence-associated genes in ML-infected armadillo liver, genes significantly up- or down-regulated in infected armadillo livers (versus control) were compared with the genes identified in the CellAge database of cell senescence genes as senescence-inducing or senescence-inhibiting.^56^

### Quantification and statistical analysis

For armadillo liver: body weight comparisons, liver enzyme assays and for western blot quantification, labelled positive cell quantification *in situ*, statistical significance was calculated using two-tailed *t-tests*, with errors bars denoting mean ± SEM. RNAseq data was processed as described, with significance in log2 transformed FPKM values of the expressed genes tested by ANOVA. Boxplots were generated by geom_boxplot in the ggplot2 package in R environment, displaying the median, first and third quartiles, with whiskers extending to the largest or smallest values no further than 1.5x the interquartile range from the third or first quartile, with outlier points beyond this plotted individually. Boxplot data was checked for assumptions allowing parametric testing, with lobule area analysis applying Kruskal-Wallis one-way ANOVA, nuclear density analysis using ANOVA with post-hoc Tukey’s.

## Data Availability

RNA-sequencing data generated in this study has been deposited to GEO and the accession code included in the key resources table. Whole slide images of H&E stained sections of liver from nine-banded armadillos chronically systemically infected by *Mycobacterium leprae*, resistant to systemic infection by *Mycobacterium leprae*, or uninfected and used in this paper (acquired on a Hamamatsu NanoZoomer in. ndpi format) are available at https://doi.org/10.7488/ds/3147. Detailed description of bespoke data analysis methods and pipelines using code are provided within the published article. All other data reported in this paper will be shared by the lead contact upon request without restriction. Any additional information required to reanalyze the data reported in this paper is available from the lead contact upon request.

## Supplemental information

Document S1. Figures S1–S10 and Tables S1 and S2

Data S1. Differentially expressed genes in ML-infected versus control armadillo liver based on cell-type-specific annotation from scRNA-seq data from adult human liver,^42^ related to Figures 3 and 4

Data S2. GO terms returned by g:GOSt analysis from g:Profiler for shared genes differentially expressed in ML-infected versus control armadillo liver and also defining EPCAM^+^ adult human liver progenitor cluster 4 from Aizarani et al.^42^ or AFP^+^ adult human progenitors from MacParland et al.,^44^related to Figures 3 and 4

Document S2. Article plus supplemental information
