# Supplementary material for: In vivo partial reprogramming by bacteria promotes adult liver organ growth without fibrosis and tumorigenesis
Source: Cell Rep Med. 2022 Nov 15;3(11):100820. doi: 10.1016/j.xcrm.2022.100820 (PMC9729881; doi:10.1016/j.xcrm.2022.100820)
Supplement: Document S1. Figures S1–S10 and Tables S1 and S2 [file mmc1.pdf]

Cell Reports Medicine, Volume *3*

Supplemental information

In vivo partial reprogramming by bacteria promotes adult liver organ growth without fibrosis and tumorigenesis

Samuel Hess, Timothy J. Kendall, Maria Pena, Keitaro Yamane, Daniel Soong, Linda Adams, Richard Truman, and Anura Rambukkana

Supplemental Fig. 1 (related to Fig 1)

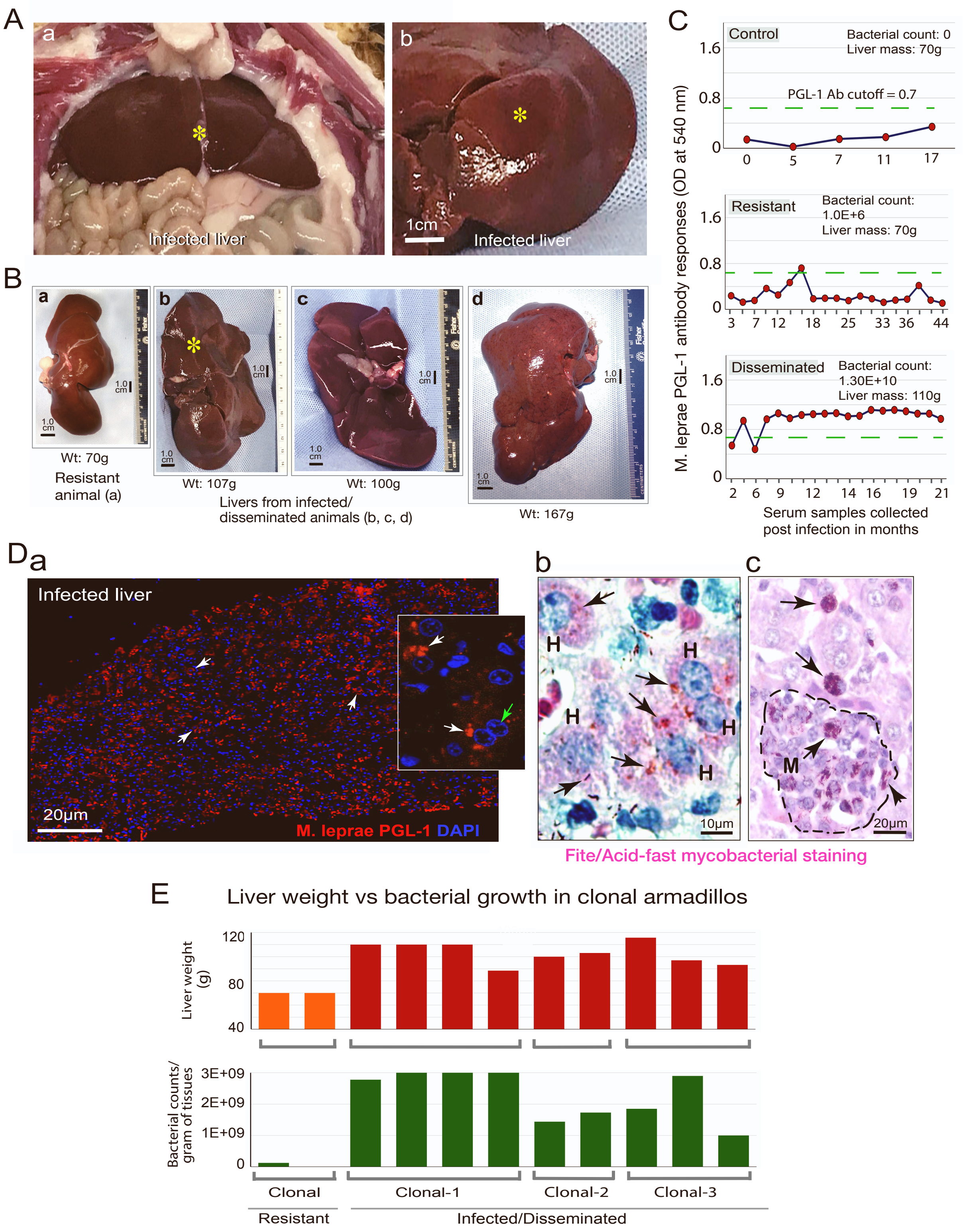

Enlarged livers in ML infected animals with hepatocellular bacterial propagation are macroscopically normally lobated
(A) Livers of armadillos with disseminated ML infection are situated within the abdomen as normal animals (A-a) and have a non-nodular, clean and smooth capsular surface, indistinguishable from uninfected/control livers, but only larger (A-b); asterisks in A-a, A-b and B-b show the same enlarged infected liver.

(B) The lobation of livers from those resistant to infection (B-a), and those with chronic disseminated infection (B-b, -c, -d) is identical although the livers of infected animals are larger (B-b, -c, -d).

(C) Representative examples of monitoring the progression of infection by serology in uninfected/control and infected animals over the indicated time period by using analysis of serum antibody response to ML specific PGL-1 antibody by ELISA, measuring optical density (OD) at 540nm. Established, disseminated infection is regarded when sustained OD540 measurements above a cut-off of

0.7 (dashed green line), are observed in successive time points, not observed in control or resistant animals; liver mass and bacterial count at time of sacrifice is shown.

(D) (D-a) Immunofluorescent of bacterial labelling of infected livers demonstrates that ML, as detected by monoclonal antibody to ML-PGL-1 (red), are distributed throughout the tissue and are exclusively located within the cytoplasm of cells with the nuclear features of classical (white arrows with spherical nuclei) and distinct binuclear hepatocytes (green arrow in the inset); DAPI labelling denotes nuclei (blue). (D-b) Fite staining that detect acid-fast (AF) mycobacteria indicates the intracellular localization of bacilli to both hepatocytes (indicated as H), non-hepatic cells (D-b) and within macrophages (M) in scattered small granulomas (dotted circled area) and other cells as shown by the arrows (also see supp Fig. 9 for in situ hepatocytes vs small granuloma distribution).

(E) Liver weight (upper) and bacterial load (lower) of genetically identical (clonal) armadillo siblings within infected-resistant and infected-disseminated animals studied, showing similar outcomes of liver growth and bacterial count within each clonal group.

Supplemental Fig. 2 (related to Fig 2)

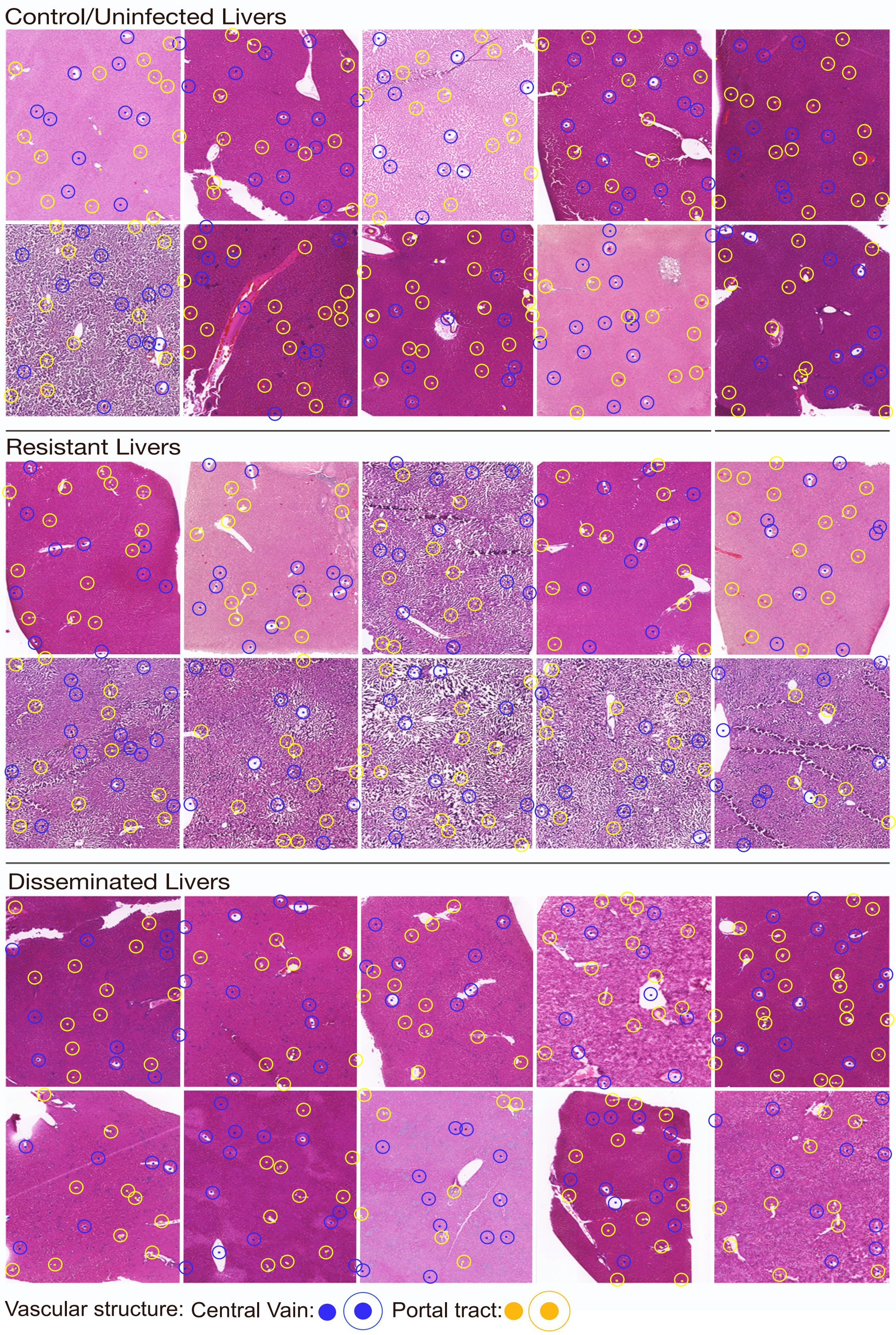

Intact vascular architecture of enlarged infected livers

Uniform regions of interest from 10 animals each from control, resistant and disseminated/infected groups with hepatic artery branches of portal tracts (yellow) and central veins (blue) are marked. Includes examples of H&E-stained sections of formalin-fixed paraffin-embedded and frozen liver tissues. Disseminated animals included were from post-infection 10-30 months.

Supplemental Fig. 3 (related to Fig 2)

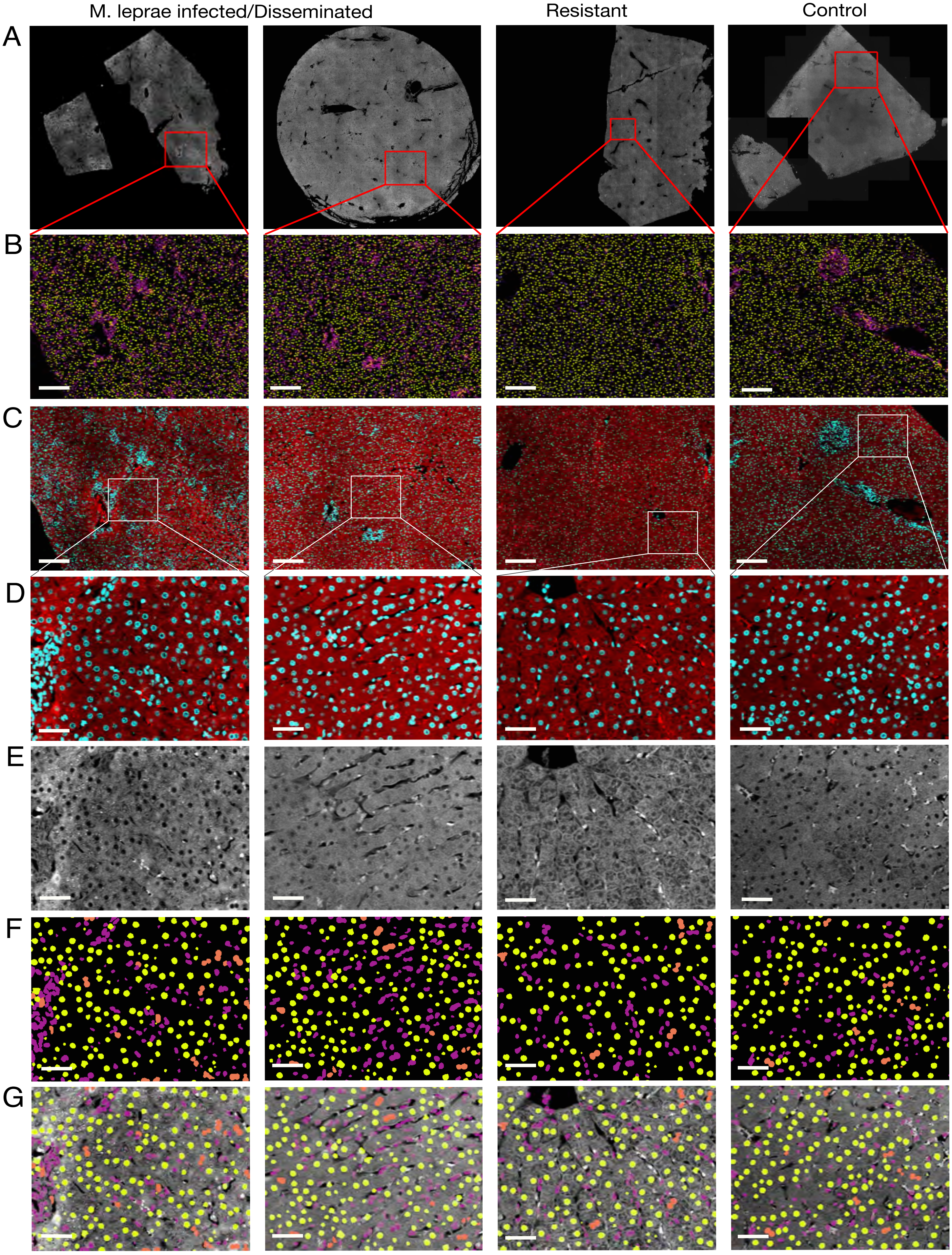

Machine-learning approach defines the intact hepatic cell population of adult armadillo Livers

Representative example entire tissue sections from Control/uninfected (top panel), Resistant (middle panel), and Disseminated (two bottom panels). Images were acquired via whole-slide scanning on a Zeiss AxioScan.Z1 using DAPI and autofluorescence (AF) channels.

(A) Overview in AF is shown in column (A) with inset region chosen for b and c in a red box. The region used was selected to represent the various tissue and cell morphologies across the whole sample.

(B) Column (B) shows computationally classified cells after application of a manually trained machine-learning decision-tree model and stepwise binuclear reclassification was applied to automatically segmented DAPI-channel nuclei. Nuclear classes are Hepatocyte (yellow), Binuclear (orange), and ‘other’ (non-parenchymal) cell types (purple).

(C) Column (C) shows the same regions as (B) but in original DAPI (blue) and AF (red) mixed fluorescence image. Intensity and texture metrics of these channels, along with morphology of the nuclear objects, were used to train the decision-tree model as described in detail in Materials and Methods.

(D) Column (D) shows the inset (white box) region from (C) in more detail where individual sinusoids and hepatocyte plates are clear.

(E-G) Column (E) shows the same regions as (D) but in AF channel only to further highlight the hepatic lobular architecture. A computationally classified image of this region can be seen in (F) and the matching overlay in (G) shows the alignment of yellow cells with hepatocyte plates and purple non-parenchymal cells within sinusoids. Scale bars: 200μm (B & C); 50μm (D-G).ipsum

Supplemental Fig. 4 (related to Fig 2, 4, 5)

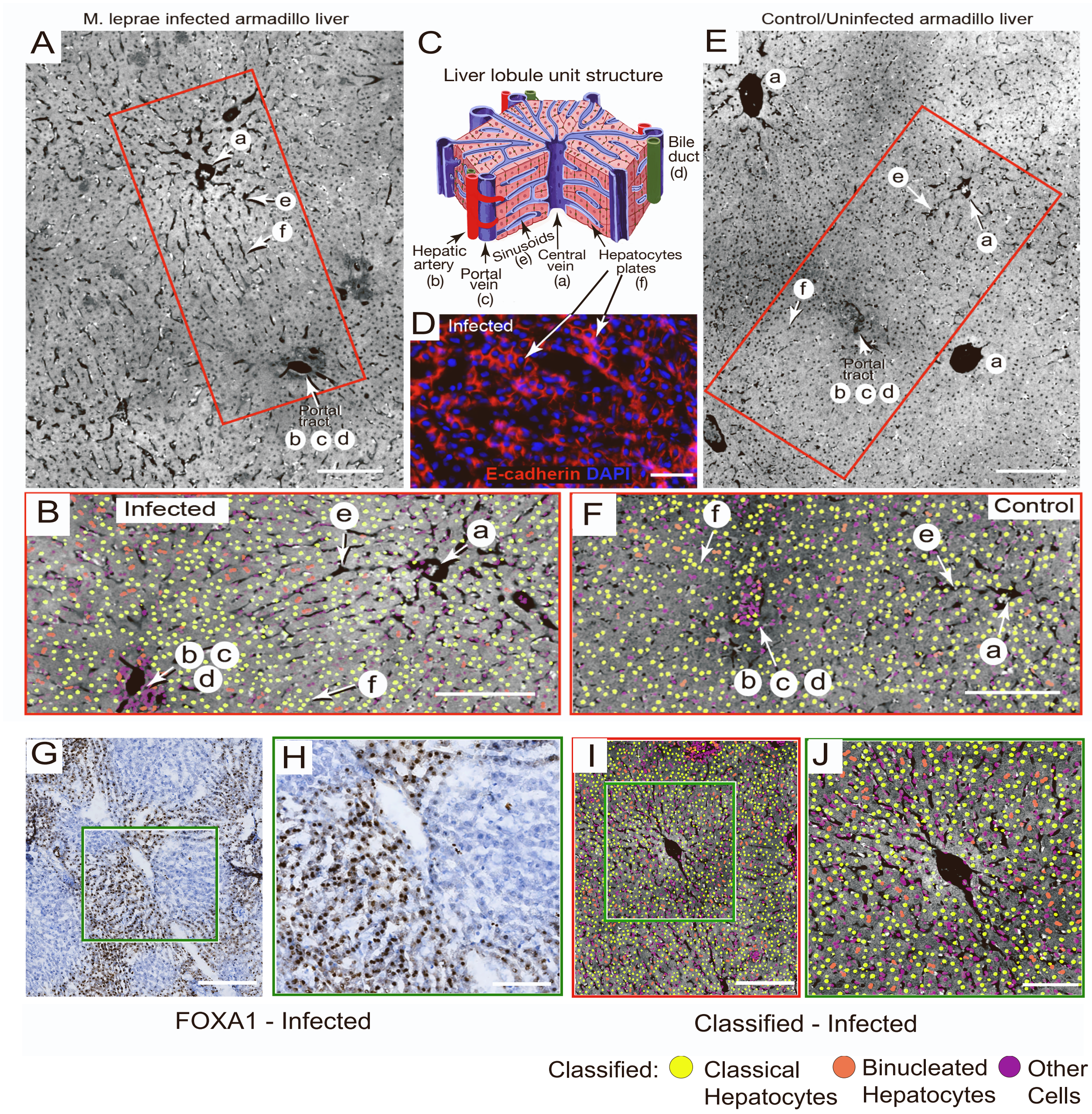

ML-infected enlarged livers demonstrate normal and intact sinusoidal and lobular architecture and reactivation of liver developmental/progenitor markers.

Representative example liver tissues from ML infected (A & B) and control uninfected animals (F & G). Images were acquired via whole-slide scanning on a Zeiss AxioScan.Z1 using DAPI and autofluorescence (AF) channels (A, B, E, F, I, J). Overview in AF is shown in (A) and (E) with inset region chosen (B and F) in a red box. The region used was selected to represent the various tissues, vessels and cell morphologies across the whole sample. Insets show computationally classified cells after a manually trained machine-learning decision-tree model and stepwise binuclear reclassification was applied to automatically segmented DAPI-channel nuclei (also see main Fig. 2). Nuclei classes are 'Hepatocyte' (yellow), 'Binuclear' (orange), and 'Other' cell type (purple). Intensity and texture metrics of these channels, along with morphology of the nuclear objects, were used to train the decision-tree model as described in detail in materials and methods. The intact hepatic lobule architecture and normal sinusoidal arrangement with hepatocyte plates, as shown in schematic (C) are evident in infected livers (A, B). Arrows with lowercase letters indicate the lobule unit structures depicted in the schematic in C. Expression of liver progenitor markers E-cadherin (D) and FOXA1 (G, H) on the membrane and nuclei of hepatocyte plates respectively is shown in 24-month infected livers. Comparison of the expression of FOXA1 (G, H) with classified hepatocytes from adjacent sections from the same 24-month infected animals as determined by signature nuclear morphology of hepatocytes computationally classified by a trained machine-learning classifier (I, J), indicating most of the FOXA1 positive cells are classical hepatocytes (yellow) within hepatic plates and thus ML infection reactivated FOXA1 protein in some of adult hepatocytes. Both FOXA1 and FOXA2 are negative in control/uninfected livers as shown in the main Figs. 5E-a; 5F-a. Scale bars are 200µm (A and E), 50µm (B, D, F) 200µm (G, I), and 100µm (H, J).

Supplemental Fig. 5 (related to Fig 2, 4, 5)

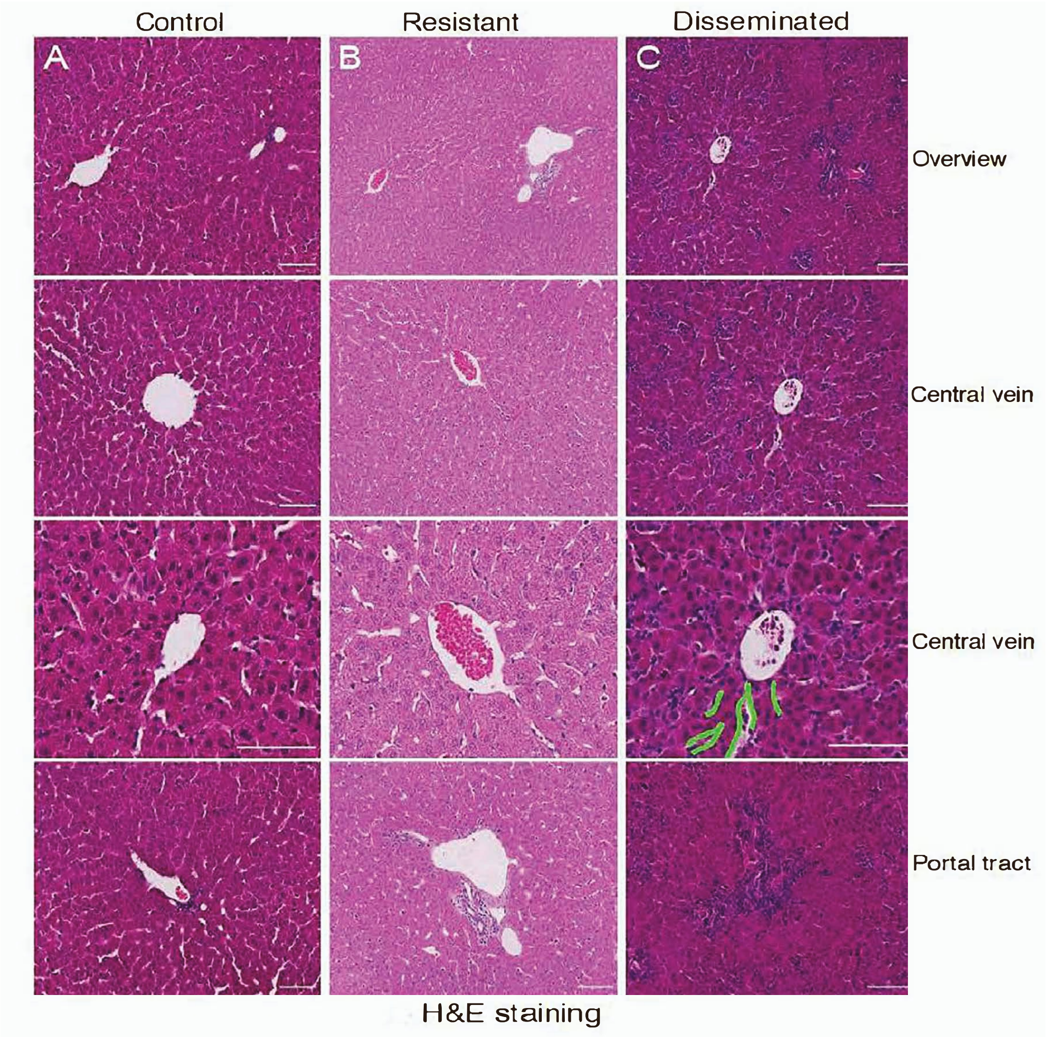

Focal hepatic cell plate twinning in an animal with disseminated infection. In livers of control and resistant animals hepatic cell plates are single (A and B). An example in one animal with disseminated infection shows that there is focal cell plate twinning (outlined in green) in pericentral areas (C), suggesting histological evidence for some regenerative activities (scale bars 100 µm).

Supplemental Fig. 6 (related to Figures 3, 4, 5, 6)

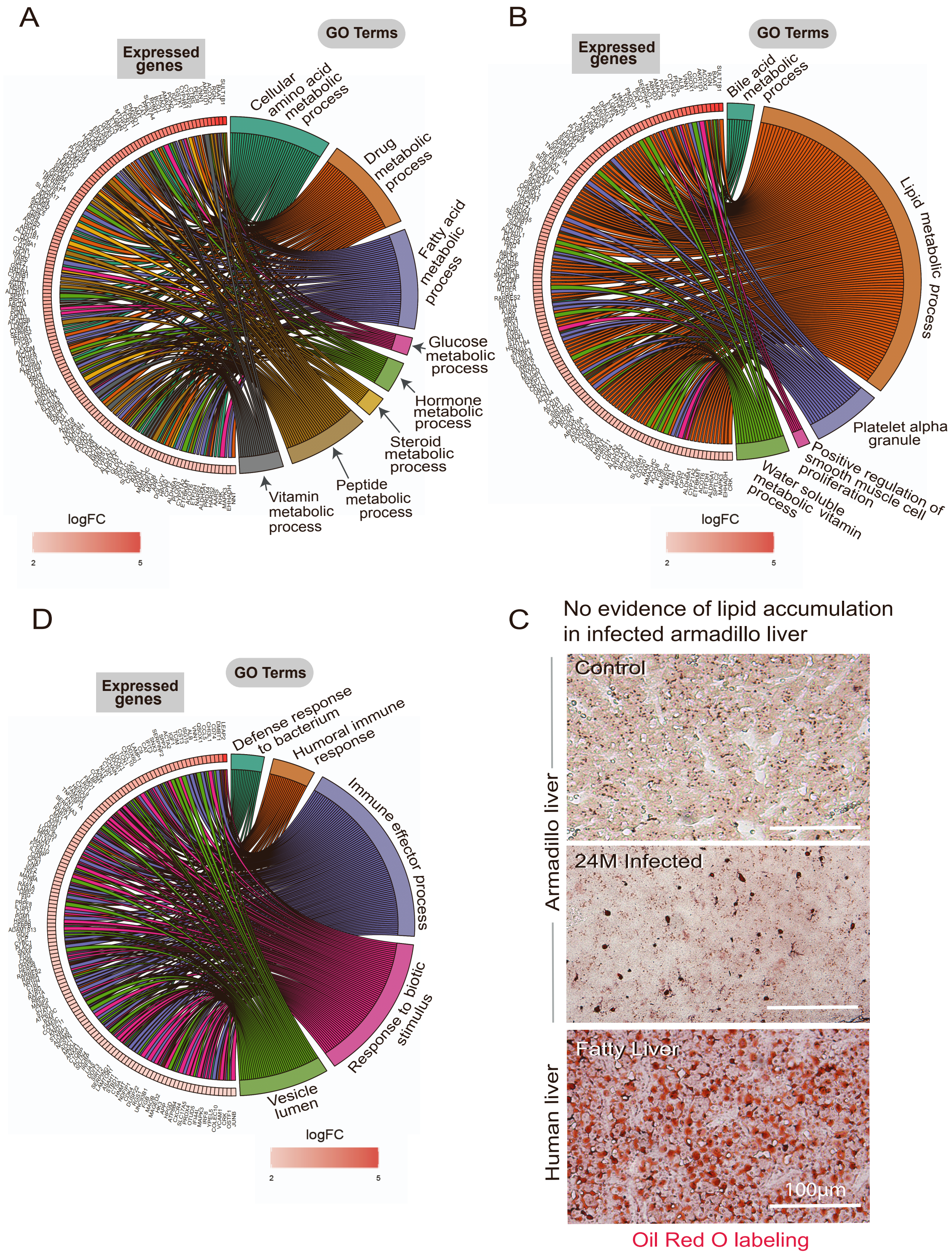

***In vivo* ML infection induces a gene transcription reflecting liver metabolic functions that are normal cellular constituents of a functional liver in addition to immune-related responses.**

(A) Chord diagrams linking up-regulated genes and companion mapped GO terms in liver infection demonstrate enhanced, broad-activity liver-related metabolic functions.

(B, C) Genes encoding lipid metabolic processes are upregulated without pathogenic hepatic steatosis as indicated by the absence of Oil Red O positive lipid in the livers of infected animals in addition to the absence of macrovesicular steatosis which is visible on Oil Red O lipid -stained sections from human fatty liver disease (C)).

(D) A chord diagram showing immune and defence responses to bacterial infection, and biliary epithelial, quiescent hepatic stellate cell, and vascular cells. These together show a response to ML coupled to gene transcription that reflects the functioning of all the normal cellular constituents and defence responses of the infected armadillo liver.

Supplemental Fig. 7 (related to Figures 3, 4)

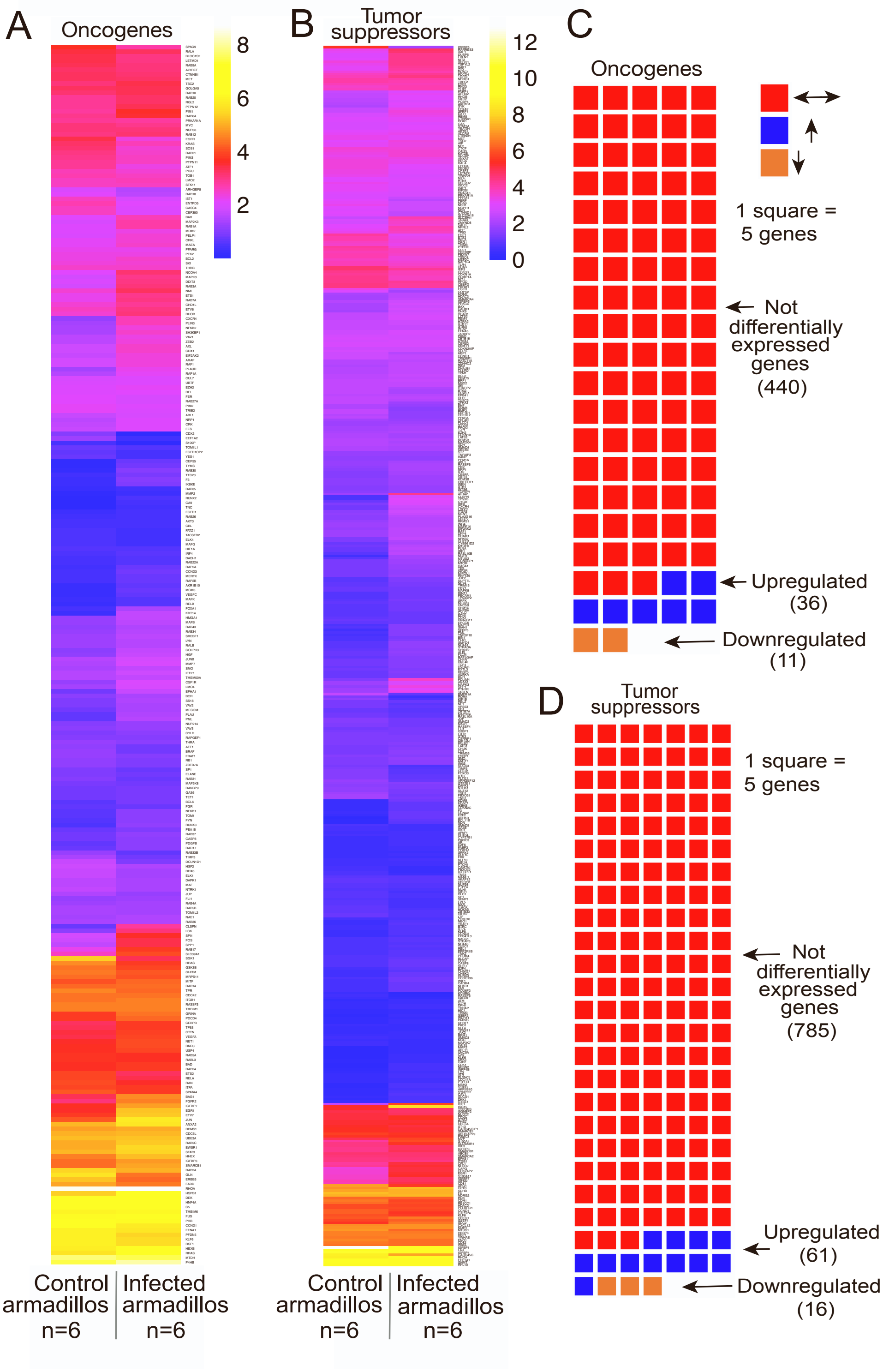

Minimal expression of oncogenes and tumor suppressor genes corroborates with the lack of histopathological evidence of tumorigenesis in infected armadillo livers

(A, B) Hierarchical clustering heatmaps on the expression of oncogenes (A) and tumor suppressor genes (B) in the Mycobacterium leprae infected and uninfected normal armadillo livers. The gene expression levels were represented by the group average of log2 (FPKM) values for six samples from each group and displayed by the colors using the color key scale on the top right. The known oncogenes and tumor suppressor genes on the heatmaps were based on Arraystar LncPath™ Cancer Microarrays collections (https://www.arraystar.com/lncpath-cancer-microarrays/; Arraystar Inc., Maryland), which were curated by knowledge-based expert review of scientific literatures.

(C, D) Waffle diagram of RNA-seq summarize these data for oncogenes (C) and tumor suppressor genes

(D) and, indicating a large majority of cancer-related factors are not differentially expressed (red) in infected livers compared to control livers, with a relatively small proportion upregulated (blue) or down-regulated (orange), and thus corroborated with the lack of histological and macroscopic evidence for tumor formation in infected livers.

Supplemental Fig. 8 (related to Figure 5, 6)

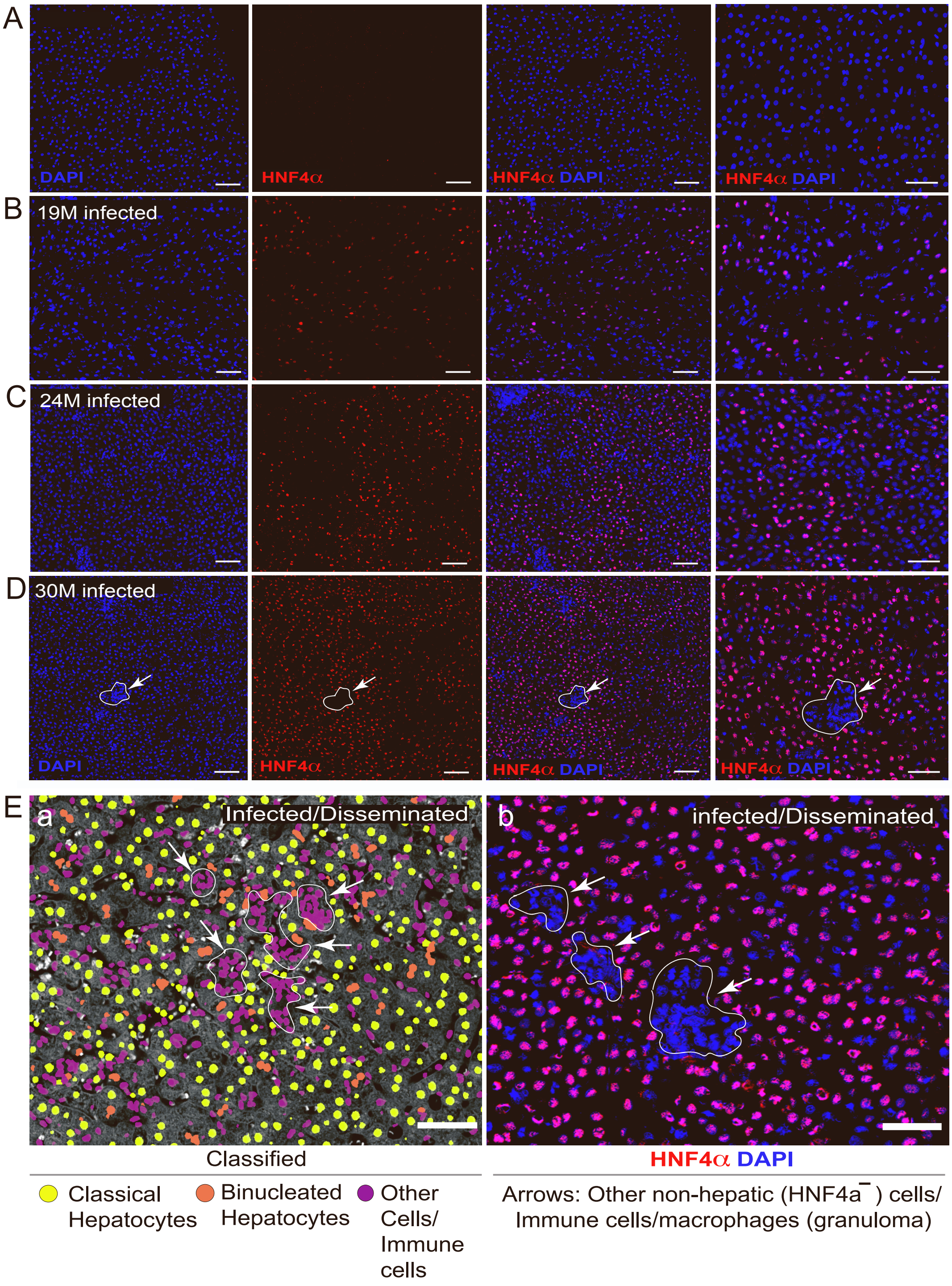

***In situ* expression of HNF4α in hepatocytes but not in small granulomas or other cells in infected armadillo livers: Correlation of hepatocyte-specific transcription factor with classified cell types by machine learning.**

(A-D) HNF4α immunoreactivity in the livers of uninfected/control (A) and disseminated armadillos after (B) 19, (C) 24 and (D) 30 months of ML infection. Note the increasing nuclear HNF4α immunopositivity over the time course of infection and throughout lobules; although HNF4α is weak in control animals western blot analysis showed clear band of HNF4α reactivity (Fig. 4A).

(E) A representative example of machine-learning-classified nuclei of infected liver showing non-hepatocyte nuclei (purple) clustered in granulomas versus non-clustered hepatocyte nuclei (yellow) in hepatic plate (E-a), as described in Star Methods and Figs 2, Suppl fig. 3. Shown here in the outlined areas are scattered small granulomas (arrows). (E-b) A section of infected/ disseminated liver showing specific HNF4α positive nuclei in hepatocytes are completely absent in small granulomas, further validating machine-learning-classification of nuclei representing classical hepatocytes vs other cells including immune/ macrophages (outlined and arrowed; also see Suppl Fig. 1-D). Scale bars: 100μm.

Supplemental Fig. 9 (related to Figure 5, 6)

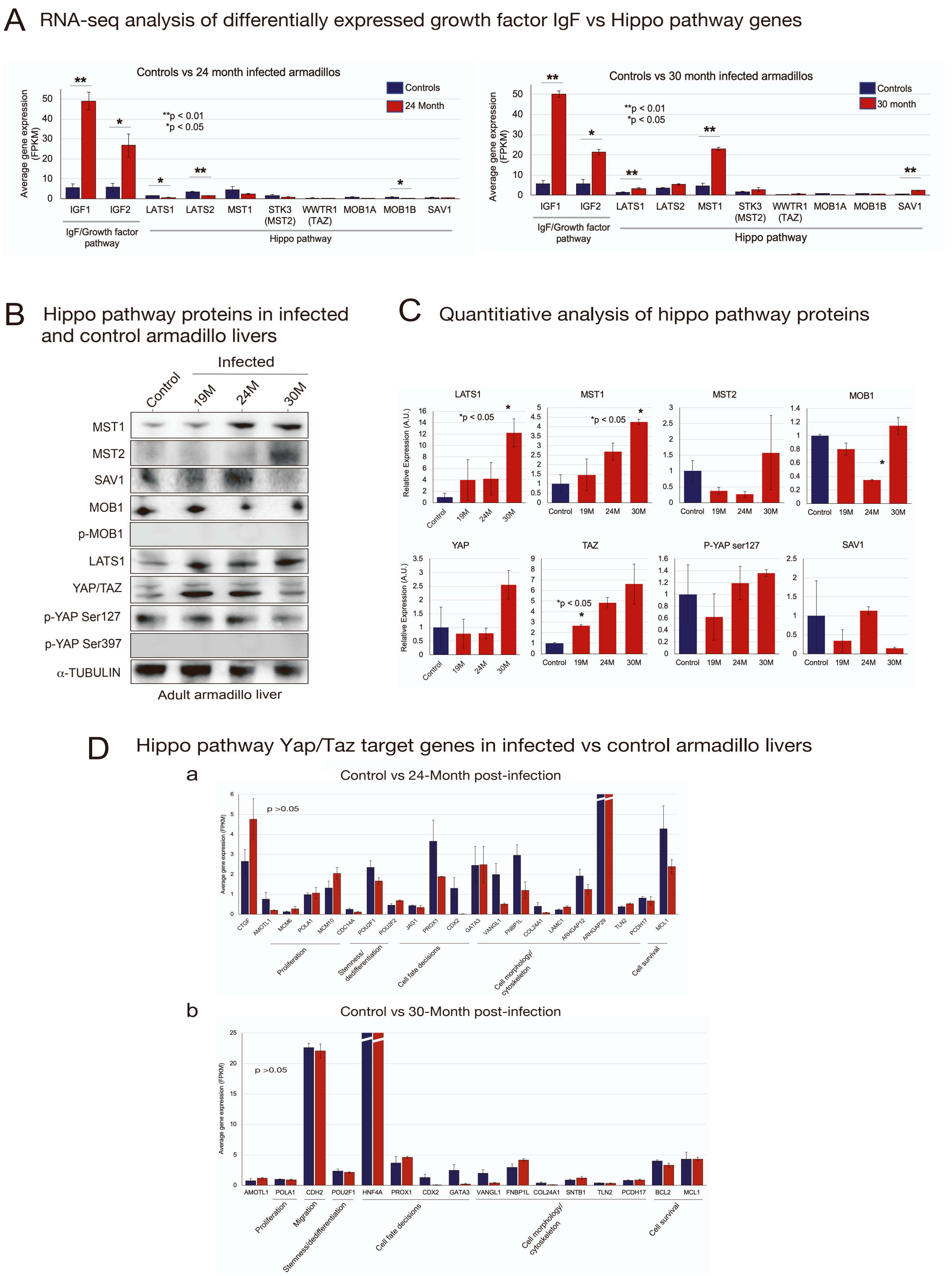

Hippo pathway components are not likely to involve in M. leprae driven armadillo liver growth.

(A) Differential expression of genes encoding growth factors IGF1 and IGF2 as compared to proteins of Hippo pathway in armadillo livers at 24- and 30-month post infection, showing markedly increased IGF1/2 level in comparison to genes in the hippo pathways.

(B) Western blot analysis of proteins and phosphorylation of proteins of hippo pathway in armadillos infected for 19, 24 and 30 months.

(C) A quantitative analysis of protein levels in control and infected animals. Note that only few hippo pathway proteins are modulated by infection at both gene and protein level and none was phosphorylated during infection.

(D) Yap/Taz target genes of various functional categories expressed in uninfected/control (blue bars) and infected armadillo livers (red bars) of post infection at 24 months (a) and 30 months (b).

Supplementary Figure 10 (related to Figure 5)

A Aminoacid sequence of the extracellular domain of CD68 from nine banded armadillos

MGKDCPHKKSATLLPSFTVTPTATESTASTATASHRTTKSHKTTSHKTTTHRTTTHQPTTHQSTTSPGPTNATHNPATTT
SHGNATVHPTSNSTTSQGTTSTSSPHPRPPPPSPSPSPGSKEAEGDYTWLNGSQPCIRLQAQIQIRVLYPTQDGEEAWGI
SVLNPNKTKAEGECGGAHAHLLLTFPYGQLSFGFKQEPTQGTVYLNYMAMEYNVSFPRTTQWTFLAENASLGDLQAPLGRSF
SCRNASIMLSPALHVDLLSLQVQAAQLPPTGVFGPSFSCPSDQGSHHHHHH

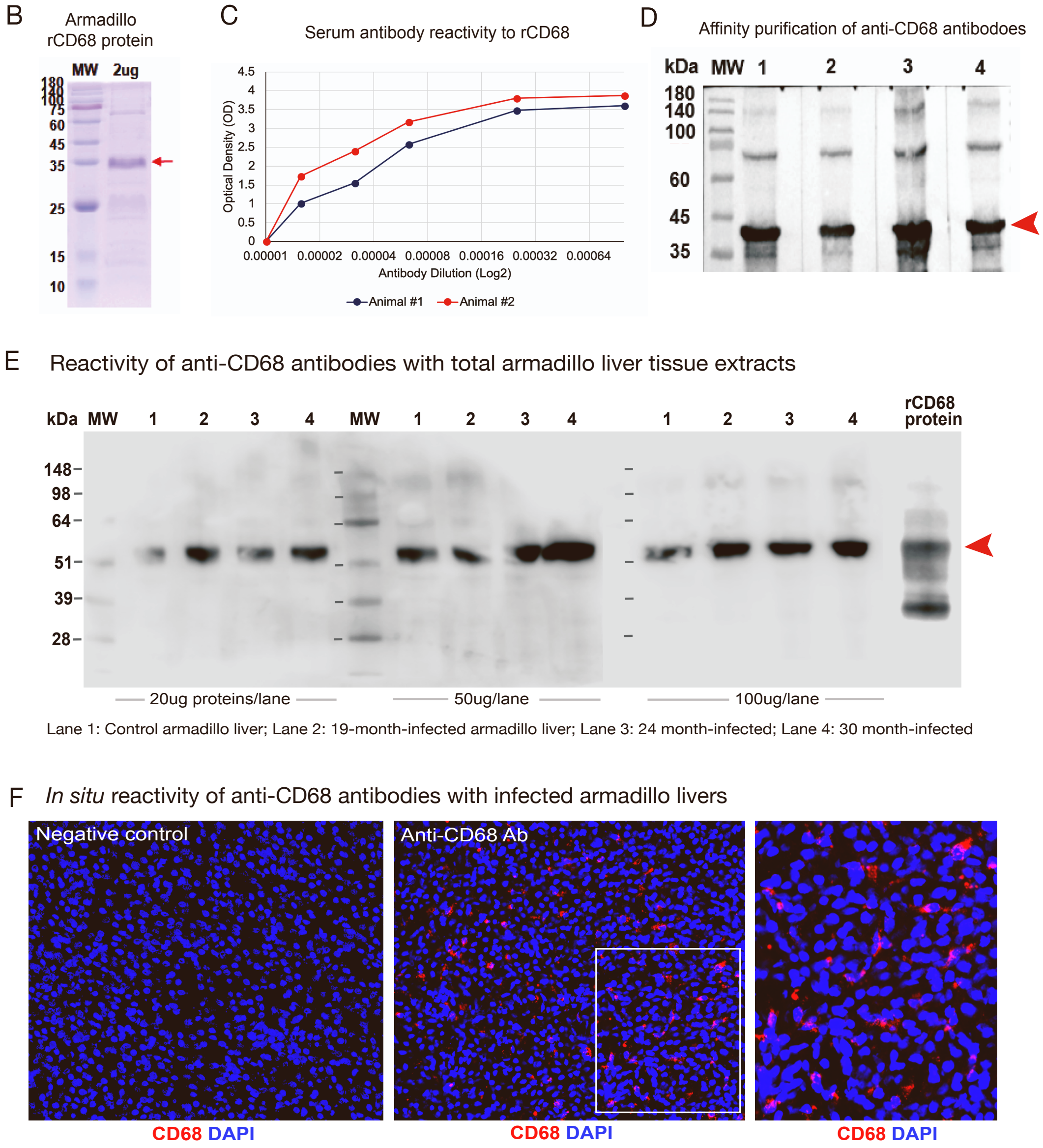

Generation and characterization of nine-banded armadillo-specific CD68 rabbit antibodies to macrophages

(a) Amino acid sequence of extracellular domain of CD68 specific for nine-banded armadillo.

(b) Commassie blue stained recombinant (r) armadillo CD68 protein (fused with His-tag) expressed in E-coli and purified from affinity column. 2ug protein of rCD68 was loaded per lane (red arrow). Purity: 75%; total protein concentration:1.3mg/ml.

(c) Antibodies were raised in rabbits against armadillo-specific rCD68 protein. Standard ELISA of serum antibody reactivities to rCD68 protein from two rabbits is shown as OD at 450nm.

(d) Western blot validation of affinity purified rabbit serum antibodies against armadillo rCD68 protein (buffer used: PBS, none-sodium azide, pH 7.4). Sera from two rabbits were affinity purified and reacted with 0.5ug of rCD68 protein at dilutions of 1:8000 and 16,000 (rabbit # 1: lane 1, 2; rabbit #2 lane 3, 4). Arrow shows the strong reactivity of purified antibodies with rCD68 fused with His-tag fusion protein.

(e) Reactivity of affinity purified anti-CD68 antibodies with total liver tissue extracts from control and infected armadillos with indicated protein concentrations.

(f) In situ reactivity of affinity purified anti-CD68 antibodies with infected armadillo liver tissues.

Supplemental Table 1 (related to Fig 1)

Details of nine-banded armadillos used in this study

Infection status	Armadillo	Gender	Wild captured/ Born in captivity	Bacterial count/ gram of tissue	Body weight (g)	Liver weight (g)	Liver:Body weight ratio	Anti-PGL-1 response (OD at 540nm)
Uninfected Control	C1	F	Captivity	N/A	2181.78	40	0.0183	N/A
	C2	F	Wild	N/A	4603.96	84	0.0182	N/A
	C3	F	Wild	N/A	5057.55	90	0.0178	N/A
	C4	M	Wild	N/A	4173.05	70	0.0168	N/A
	C5	F	Wild	N/A	5397.75	90	0.0167	N/A
	C6	M	Wild	N/A	5647.23	91	0.0161	N/A
	C7	M	Wild	N/A	4808.08	75	0.0156	N/A
	C8	F	Wild	N/A	5481.30	70	0.0128	N/A
	C9	M	Captivity	N/A	ND	ND	ND	N/A
	C10	M	Wild	N/A	ND	ND	ND	N/A
	C11	F	Captivity	N/A	ND	ND	ND	N/A
	C12	F	Wild	N/A	ND	ND	ND	N/A
Infected Resistant	R1	M	Captivity	3.66E+06	5102.91	101	0.0198	0.216
	R2	M	Captivity	3.41E+06	5012.20	90	0.0180	0.473
	R3	F	Captivity	5.99E+05	4581.28	90	0.0196	0.18
	R4	F	Captivity	3.57E+06	3742.14	59.4	0.0159	0.617
	R5	F	Captivity	1.22E+08	4014.29	70	0.0174	0.562
	R6	F	Captivity	1.03E+06	4082.33	70	0.0171	0.11
	R7	F	Wild	8.65E+04	5375.07	100	0.0186	0.007
	R8	F	Wild	2.33E+05	5397.75	90	0.0167	0.263
	R9	F	Wild	6.55E+06	5624.55	90	0.0160	0.328
	R10	F	Captivity	3.30E+02	3764.82	ND	ND	0.195
	R11	F	Captivity	4.70E+02	4944.16	ND	ND	0.571
	R12	F	Captivity	7.42E+02	3787.50	ND	ND	0.236
	R13	M	Wild	ND	5851.34	ND	ND	0.452
Infected Disseminated	D1	M	Captivity	2.78E+09	3628.74	110	0.0303	0.644
	D2	M	Captivity	2.19E+11	3832.86	110	0.0287	0.789
	D3	M	Captivity	1.04E+10	3923.57	110	0.0280	1.486
	D4	M	Captivity	2.28E+10	3742.14	88.4	0.0236	1.123
	D5	M	Captivity	1.44E+09	3197.80	100	0.0313	0.507
	D6	M	Captivity	1.73E+09	3311.20	103	0.0311	1.026
	D7	F	Captivity	1.85E+09	4280.85	115.7	0.0270	0.751
	D8	F	Captivity	2.90E+09	3782.55	97	0.0256	1.071
	D9	F	Captivity	1.00E+09	3963.75	93.2	0.0235	1.089
	D10	M	Wild	2.53E+11	3356.58	180	0.0536	1.072
	D11	M	Wild	3.96E+09	6010.10	290.32	0.0483	1.05
	D12	F	Captivity	1.37E+09	3175.15	110	0.0346	0.555
	D13	F	Captivity	1.51E+10	2698.87	80	0.0296	0.516
	D14	F	Wild	1.52E+08	3814.71	110	0.0288	0.289
	D15	F	Wild	2.62E+08	4937.70	139.2	0.0282	1.148
	D16	F	Wild	1.14E+09	6274.05	167.2	0.0266	1.132
	D17	F	Captivity	2.20E+11	3764.80	100	0.0266	1.614
	D18	F	Wild	6.70E+10	4983.00	125.9	0.0253	1.102
	D19	F	Captivity	1.44E+09	3583.38	90	0.0251	1.307
	D20	F	Captivity	1.78E+10	3311.22	80	0.0242	0.585
	D21	M	Captivity	1.00E+09	3850.50	87.3	0.0227	1.206
	D22	M	Wild	8.72E+08	5164.20	115.2	0.0223	1.068
	D23	F	Captivity	1.21E+08	3397.50	75.2	0.0221	1.184
	D24	F	Wild	1.14E+10	4575.30	100.8	0.0220	0.945
	D25	M	Wild	9.42E+07	5084.77	111.9	0.0220	1.022
	D26	F	Wild	1.30E+10	5102.91	110	0.0216	0.972
	D27	F	Wild	3.12E+11	5307.03	110	0.0207	1.745
	D28	M	Wild	3.03E+09	6622.45	137.1	0.0207	1.031
	D29	M	Wild	2.04E+08	4620.60	94.3	0.0204	1.164
	D30	F	Captivity	2.26E+10	4059.65	80	0.0197	0.963
	D31	F	Wild	3.92E+08	5420.40	100	0.0184	1.776
	D32	M	Wild	1.80E+08	4937.70	68.9	0.0140	1.252

ND: No data available

Supplemental Table 2 (related to Fig 2)

Supporting details for characterization of liver cellular composition with manually trained machine-learning based analysis of DAPI labelled liver samples.

A: *Model Parameters*

Morphological metrics	Intensity metrics	
Border index	Histogram maximum AutoFL	Circular StdDev/Mean AutoFL
Area	Histogram maximum DAPI	Circular StdDev/Mean DAPI
Roundness	StdDev. to neighbor pixels AutoFL	mode[Maximum] AutoFL
Compactness	StdDev. to neighbor pixels DAPI	mode[Maximum] DAPI
Shape index	Skewness AutoFL	mode[Median] AutoFL
Radius of largest enclosed ellipse	Skewness DAPI	mode[Median] DAPI
Length/Width	Max. pixel value AutoFL	mode[Minimum] AutoFL
Rectangular Fit	Max. pixel value DAPI	mode[Minimum] DAPI
Radius of smallest enclosing ellipse	Mean AutoFL	Min. pixel value AutoFL
Density	Mean DAPI	Min. pixel value DAPI
Elliptic Fit	Standard deviation AutoFL	Edge Contrast of neighbor pixels AutoFl
Asymmetry	Standard deviation DAPI	Edge Contrast of neighbor pixels DAPI
Circularity	Circular StdDev AutoFL	MaxDAPI
Circularity ratio	Circular StdDev DAPI	MinDAPI
Ellipticity	Mean of inner border AutoFL	Border Contrast AutoFL
	Mean of inner border DAPI	Border Contrast DAPI
	Mean of outer border AutoFL	Contrast to neighbor pixels AutoFL
	Mean of outer border DAPI	Contrast to neighbor pixels DAPI

B: *Stepwise binuclear reclassification pipeline*

Hepatocyte nuclei with 3 or more touching non-hepatocyte neighbours → non-hepatocyte
Hepatocyte nuclei with relative border ≥ 5% to another hepatocyte nucleus → binuclear
Hepatocyte nuclei touching a single nucleus of any type and with relative border ≥ 20% → binuclear
Non-hepatocytes touching binuclear with relative border ≥ 15% → binuclear
Hepatocyte nuclei with Area > 75ɼ m^2 and Circularity < 6.6 → binuclear
Hepatocyte nuclei with Area > 80ɼ m^2 and Circularity < 7 → binuclear
Hepatocyte nuclei with Area > 90ɼ m^2 and Ellipticity ≤ 0.75 → binuclear
Hepatocyte nuclei with Area > 100ɼ m^2 and Circularity < 7.5 → binuclear

C: *Error Matrix*

	PPV	NPV	Sensitivity	Specificity	Accuracy	FDR	FNR	FOR
**Training population**	0.9668	0.9165	0.9404	0.953	0.9455	0.0332	0.0596	0.0835
**Test Population**	0.9239	0.8846	0.9274	0.8794	0.9088	0.0761	0.0726	0.1154
**Combined Populations**	0.9534	0.9075	0.9364	0.9316	0.9345	0.0466	0.0636	0.0925
**Binuclear**	0.8365	0.9884	0.722	0.9941	0.9832	0.1411	0.1635	0.278

(PPV = positive predictive value, NPV = Negative Predictive Value, FDR = False discovery rate, FNR = False Negative Rate, FOR = False Omission Rate)
